# Supplementary material for: Reformulating lipid nanoparticles for organ-targeted mRNA accumulation and translation
Source: Nat Commun. 2024 Jul 5;15:5659. doi: 10.1038/s41467-024-50093-7 (PMC11226454; doi:10.1038/s41467-024-50093-7)
Supplement: Supplementary file 1 — Supplementary Information [file 41467_2024_50093_MOESM1_ESM.pdf]

Supplementary Information

Reformulating lipid nanoparticles for organ-targeted mRNA accumulation and translation

Kexin Su[1], Lu Shi[1,2], Tao Sheng[1], Xinxin Yan[1], Lixin Lin[1], Chaoyang Meng[3], Shiqi Wu[1,2], Yuxuan Chen[1], Yao Zhang[1], Chaorong Wang[1], Zichuan Wang[1], Junjie Qiu[1], Jiahui Zhao[1], Tengfei Xu[1], Yuan Ping[1,2*], Zhen Gu[1,2*], Shuai Liu[1,2,4*]

1. State Key Laboratory of Advanced Drug Delivery and Release Systems, College of Pharmaceutical Sciences, Zhejiang University, Hangzhou, China.
2. Liangzhu Laboratory, Zhejiang University, Hangzhou, China.
3. Department of Hepatobiliary and Pancreatic Surgery, The First Affiliated Hospital, School of Medicine, Zhejiang University, Hangzhou, China.
4. Eye Center, The Second Affiliated Hospital, School of Medicine, Zhejiang University, Hangzhou, China.

*Correspondence to: pingy@zju.edu.cn; guzhen@zju.edu.cn; shuailiu@zju.edu.cn

Table of content

Supplementary Methods 2
Supplementary Figures 6
Supplementary References 33

Supplementary Methods

Lipid fusion by FRET analysis

The fusion of LNPs with endosomal mimicking liposomes was assessed by the FRET assay[1]. The FRET probes, NBD-PE and Rho-PE, were formulated in the endosome-mimicking nanoparticles, and the NBD fluorescence was diminished due to the FRET to rhodamine. When the lipid fusion occurred, the increased distance between the two probes would lead to an NBD signal increase. Endosomal mimics were prepared by mixing DOPC/DOPS/DOPE/Rho-PE/NBD-PE (molar ratio of 25/25/48/1/1) in chloroform. The thin lipid films were obtained by rotary evaporation, followed by vacuum drying for 2 h to remove the solvents. Subsequently, 1× PBS buffer (pH 7.4) was added to the dried lipid films and sonicated for 30 min, giving a total lipid concentration of 1 mM. nAcx-Cm LNPs were prepared with a total lipid concentration of 1 mM, and were formulated at nAcx-Cm/DOPE/cholesterol/DMG-PEG2000 molar ratio of 15/20/25/2. Black 96-well plate wells were spiked with 100 μL of 1× PBS buffer at pH 5.5 and pH 7.4, respectively. Afterwards, 1 μL of anionic endosomal mimicking liposomes and 10 μL of nAcx-Cm LNPs were added into the wells. Post incubation at 37 °C for 5 min, the fluorescence (F) was measured at Ex/Em of 465/520 nm on a microplate reader. Anionic endosomal liposomes in 1× PBS were set as the negative control (F_{min}). The lipid mixtures containing FRET probes and Triton X-100 (1 wt.%) were set as the positive control (F_{max}). Lipid fusion rates (%) were calculated as $(F\text{-}F_{min})/(F_{max}\text{-}F_{min})$ ×100%.

Endosomal escape and cellular uptake assay

Endosomal escape and cellular uptake of LNPs were determined by the confocal imaging. IGROV1 cells were inoculated into glass-bottomed/confocal culture dishes at a density of 200,000 cells/well. After 24 h, 1 mL of fresh medium (10% FBS) was added and the cells were treated with LNPs containing 500 ng Cy5-mRNA. LNPs were formulated with nAcx-Cm /DOPE/Cholesterol/DMG-PEG2000 molar ratio of 15/20/25/2. 4 h later, cells were washed three times with 1× PBS. The cells were stained with Lysotracker Green DND26 (1/8000 dilution) for 40 min at 37 °C, and then stained with Hoechst 33342 (0.1 mg/mL) for 5 min. Finally, the cells were imaged by the confocal microscopy.

LNP dissociation by FRET assay

The dissociation of nAcx-Cm LNPs was determined by mixing 6Ac1-C12 LNPs with the endosomal mimicking anionic liposomes. The DOPE-derived FRET probes NBD-PE and Rho-PE were prepared into the same LNPs. The specific formulation used 6Ac1-C12/DOPE/Cholesterol/DMG-PEG2000/NBD-PE/Rho-PE (molar ratio 15/20/25/2/0.63/0.63) lipid mixture to prepare the 6Ac1-C12 LNPs, with a total lipid concentration of 1 mM. The lipid mixture comprising DOPS/DOPC/DOPE with a molar ratio of 25/25/50 was formulated to get the endosomal mimicking anionic liposomes. Briefly, the liposomes in chloroform were subjected to rotary evaporation and vacuum drying for 2 h to obtain the lipid films. Subsequently, the dried films were then hydrated by sonication in 1× PBS buffer (pH 7.4) for 30 min to give a total lipid concentration of 10 mM. The 1× PBS buffer at pH 5.5 (100 μL/well), 1 μL of 6Ac1-C12 LNPs and 1 μL of endosomal mimicking anionic liposomes were added to the black 96 well plates. Following incubation at 37 °C for 5 min or other time intervals, the

fluorescence (F) was measured on a microplate reader at Ex/Em of 465/520 nm. The negative control (F_{min}) was set as 6Ac1-C12 LNPs incorporating the FRET probes in 1× PBS buffer. The positive control (F_{max}) was set as 6Ac1-C12 LNPs containing FRET probes incubated with Triton X-100 solutions (1 wt.%). The dissociation of LNPs (%) was calculated as ($F-F_{min}$)/($F_{max}-F_{min}$) ×100%.

mRNA release assay

The mRNA release of nAcx-Cm LNPs was determined by mixing 6Ac1-C12 LNPs with endosomal mimicking anionic liposomes. The 6Ac1-C12 LNPs contained 6Ac1-C12/DOPE/Cholesterol/DMG-PEG2000 with a molar ratio 15/20/25/2 (total lipid concentration, 1 mM). The endosomal mimicking lipid mixture was formulated at DOPS/DOPC/DOPE molar ratio of 25/25/50 in chloroform. The anionic lipid films were obtained by rotary evaporation followed by vacuum drying for 2 h to remove the solvents. Afterwards, the lipid film was hydrated by sonication in 1× PBS to give a total lipid concentration of 10 mM. 1 μL of 6Ac1-C12 LNPs and 1 μL of endosomal mimicking anionic liposomes were mixed, incubating at 37 °C for 5 min or 30 min. The mRNA release was detected by Quant-iT Ribogreen RNA assay kit using the microplate reader.

pK_a determination using the 2-(p-toluidino)-6-naphthalenesulfonic acid (TNS) assay

The pK_a of the LNPs was determined by TNS assay. LNPs were comprised of synthetic ionizable lipid/DOPE/cholesterol/DMG-PEG2000 (molar ratio 15/20/25/2) in the 1× PBS buffer with a total lipid concentration of 1 mM. The TNS was diluted to a 100 μM solution using milliQ water. Each well of the black 96-well plates was spiked with 100 μL of buffer solution containing 10 mM 4-(2-hydroxyethyl)-1-piperazineethanesulfonic acid (HEPES), 10 mM 4-morpholineethanesulfonic acid (MES), 10 mM ammonium acetate, and 130 mM sodium chloride (NaCl) with a pH range of 2.5 to 11. The LNPs were diluted to a final 10 μM total lipid concentration in the wells. The TNS solution was added to each well, making a final concentration of 2 μM. The plates were read using a microplate reader at Ex/Em of 321/445 nm. The fluorescence data was analyzed by a sigmoidal fit analysis and the pK_a was determined as the pH at half of the maximum fluorescence intensity.

Hydrolysis of ionizable lipid 6Ac1-C12

Lipid hydrolysis analysis was conducted as per the literature[2]. 6Ac1-C12 lipid (177.4 mg, 0.10 mmol) was added to a 50 mL round bottom flask. Next, 10 mL of 6 M HCl aqueous solution was added to give a cloudy non-homogeneous dispersion. Reaction was heated and refluxed for 24 h to obtain a clear solution. Then the mixture solution was cooled and neutralized with 4 M NaOH aqueous solution. Chloroform was utilized for extraction, and the combined organic solution was concentrated to obtain a degradable mixture of 6Ac1-C12 byproducts. The degradation mixture was analyzed by ^{1}H NMR.

mRNA-LNP accumulation assay by the flow cytometry

Flow cytometry was used to determine the accumulation of nanoparticles in different cell types. 5Ac1-C12 or 6Ac1-C12 Liver LNPs were formulated at ionizable lipid/DOPE/cholesterol/DMG-PEG2000 molar ratio of 15/20/25/2. For 6Ac1-C12 Lung LNPs,

3-, 4-, and 5-Comp Lung LNPs were formulated at ionizable lipid/DOTAP/DMG-PEG2000 molar ratio of 60/60/1.2, ionizable lipid/DOTAP/cholesterol/DMG-PEG2000 molar ratio of 60/60/40/1.2, and ionizable lipid/DOPE/cholesterol/DMG-PEG2000/DOTAP molar ratio of 15/20/25/2/62, respectively. Cell isolation and staining were performed 2 h post i.v. administration of these LNPs containing Cy5-mRNA (0.5 mg kg^{-1}) to C57BL/6 mice.

For the isolation of mouse liver cells, differential centrifugation was employed[3]. The mice were anesthetized with isoflurane and fixed on the foam plates. The mouse liver was perfused, and then clipped and digested with collagenase IV at 37 °C for 30 min. Following termination of digestion, liver cells were passed through a 70 μm cell filter and washed with 1× PBS. Subsequently, hepatic parenchymal cells were collected by centrifugation (4 °C, 50 g, 5 min), and the cell sediment was resuspended in the washing medium and then washed with 1× PBS. The supernatants were collected and the liver nonparenchymal cells were obtained by centrifugation (4 °C, 450 g, 5 min). These cells were resuspended in the red blood cell (RBC) lysis buffer and incubated for 5 min. The RBC lysis was terminated by the addition of 1× PBS. The mixture was centrifuged, counted and then resuspended in the cell staining buffer. Antibodies for staining were added into the cell suspension and incubated on ice for 30 min in the dark. After staining, the cells were washed twice with 1× PBS and finally resuspended in 500 μL of 1× PBS. Afterwards, the cell suspensions were transferred to the flow tube and analyzed with the multicolor analytical flow cytometer (LSR Fortessa, BD Biosciences). Antibodies used here included PerCP/Cyanine5.5 anti-mouse CD45 (1/200 dilution, Biolegend, 157208), PE/Cyanine7 anti-mouse CD31 (1/200 dilution, Biolegend, 102524), FITC anti-mouse/ human CD11b (1/100 dilution, Biolegend, 101205), and Brilliant Violet 785™ anti-mouse F4/80 (1/100 dilution, Biolegend, 123141). Sytox™ Blue Dead Cell Stain (1/2000 dilution, Thermo Fisher, S34857) was employed to differentiate the live cells.

To perform isolation of lung cells, mouse lungs were minced in plates and then transferred to the 15 mL centrifuge tubes containing collagenase I and DNase I digestion medium. The mixture was digested by shaking at 37 °C for 1 h in a constant temperature shaker. Post termination of digestion, the mixture was passed through a 70 μm cell filter and washed with 1× PBS. Pulmonary cells were collected by centrifugation (4 °C, 450 g, 5 min). Antibodies used here included PerCP/Cyanine5.5 anti-mouse CD45 (1/200 dilution, Biolegend, 157208), PE/Cyanine7 anti-mouse CD31 (1/200 dilution, Biolegend, 102524), and FITC anti-mouse CD326 (Ep-CAM) (1/100 dilution, Biolegend, 118207). Sytox™ Blue Dead Cell Stain (1/2000 dilution, Thermo Fisher, S34857) was used to differentiate the live cells. Ultimately, lung cells were analyzed using the LSRForessa machine. Data were analyzed using FLOWJO software version 10.8 (FLOWJO)

Isolation of protein coronas adsorbed on LNPs in plasma

6Ac1-C12 Liver and Lung LNPs were prepared and diluted to a total lipid concentration of 5 mg/mL with 1× PBS. Different LNPs were co-incubated with mouse plasma for 60 min at 37 °C. The mixture was then centrifuged at 15,000 g for 30 min at 4 °C. The supernatant was removed and the pellet was washed with 1× PBS. The washing process was conducted for a total of 3 times. The obtained plasma protein samples were stored at -20 °C for further analysis. The same procedure was conducted with plasma/PBS to certify the absence of plasma precipitation by centrifugation.

Mass spectrometry analysis of plasma protein samples

The plasma protein samples were treated with 10 mM tris(2-carboxyethyl)phosphine, 25% trifluoroethanol (v/v), and 100 mM triethylammonium bicarbonate, incubating at 55 °C for 20 min for reduction. The samples were then alkylated by 50 mM 2-chloroacetamide (CAA). Subsequently, 0.4 μg LysC was added into the samples and incubated at 37 °C for 2 h, following by addition of 1.6 μg trypsin and incubation at 37 °C overnight. Upon digestion completion, the samples were transferred to tubes with 0.5 mL of acetic acid (0.5%, v/v), and the enzymes were inactivated by trifluoroacetic acid (1%, v/v). Afterwards, another desalting step was conducted with an Oasis HLB cartridge (1 cc, WAT09422, Waters Corp.), and the solution was dried with vacuum centrifugation.

The obtained samples were re-solubilized by mobile phase A (2% acetonitrile and 0.1% formic acid), centrifuged at 20,000 g for 30 min, and loaded onto a silica capillary column employing the EASY nLC1200 nanoLC system (Thermo Scientific). Separation was conducted by a 90-min gradient elution using mobile phase A and mobile phase B (0.1% formic acid in 98% acetonitrile). The samples were further analyzed by the nanospray ionization (NSI) source, following by tandem mass spectrometry (MS/MS) in the Orbitrap Exploris™ 480 mass spectrometer combined with a FAIMS Pro™ interface (Thermo Scientific) coupled online to the UPLC. MS/MS data was processed utilizing the Thermo Scientific™ Proteome Discoverer™ 2.4 software.

Supplementary Figures

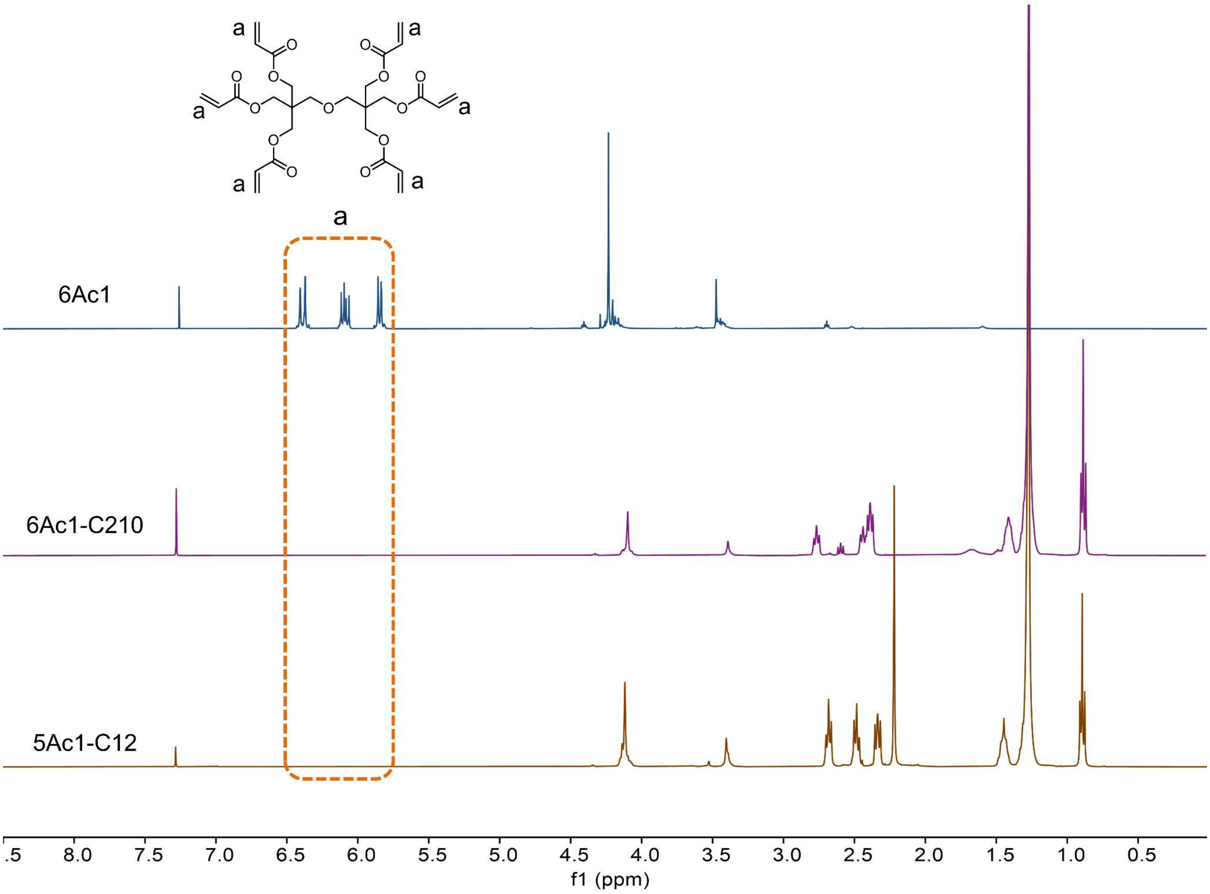

Supplementary Fig. 1 | ^{1}H NMR spectra of 6Ac1-C210 and 5Ac1-C12 in $CDCl_3$. Equal equivalent of acrylate monomer was reacted with the amine, and the disappeared peak of vinyl group (at around 5.8~6.4 ppm) indicated the complete reaction.

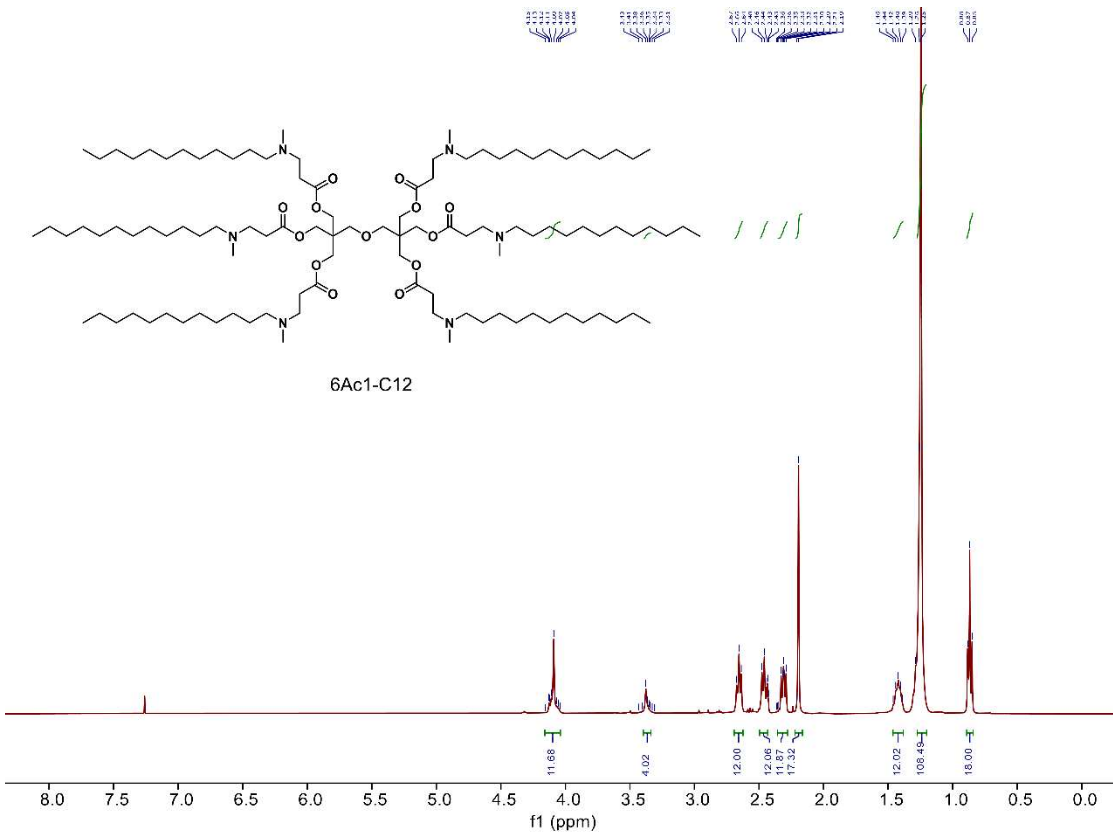

Supplementary Fig. 2 | ^{1}H NMR spectra of 6Ac1-C12 in $CDCl_3$.

^{1}H NMR ($CDCl_3$, ppm) δ 0.87 (t, 18H, -$NCH_2CH_2(CH_2)_9C\boldsymbol{H}_3$), 1.20-1.30 (m, 108H, -$NCH_2CH_2(C\boldsymbol{H}_2)_9CH_3$), 1.38-1.47 (m, 12H, -$NCH_2C\boldsymbol{H}_2(CH_2)_9CH_3$), 2.19 (s, 18H, -$NC\boldsymbol{H}_3$), 2.31 (t, 12H, -C(O)$C\boldsymbol{H}_2CH_2$N-), 2.42-2.50 (m, 12H, -$NC\boldsymbol{H}_2CH_2(CH_2)_9CH_3$), 2.66 (t, 12H, -C(O)$CH_2C\boldsymbol{H}_2$N-), 3.38 (s, 4H, -$OC\boldsymbol{H}_2C(CH_2)_3$-), 4.09 (s, 12H, -C(O)$OC\boldsymbol{H}_2$C-). MS (MALDI-TOF, m/z) Calc. for $C_{106}H_{208}N_6O_{13}$: 1773.58, found M^{+1}: 1774.56.

Levels	Factors (molar ratio)			
	6Ac1-C12	DOPE	Choles terol	DMG-PEG2000
1	10	10	15	1
2	15	15	20	2
3	20	20	25	3
4	25	25	30	4

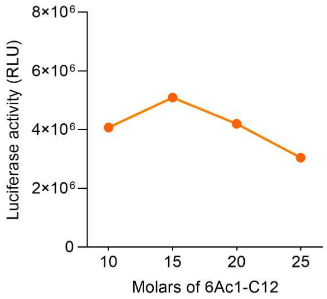

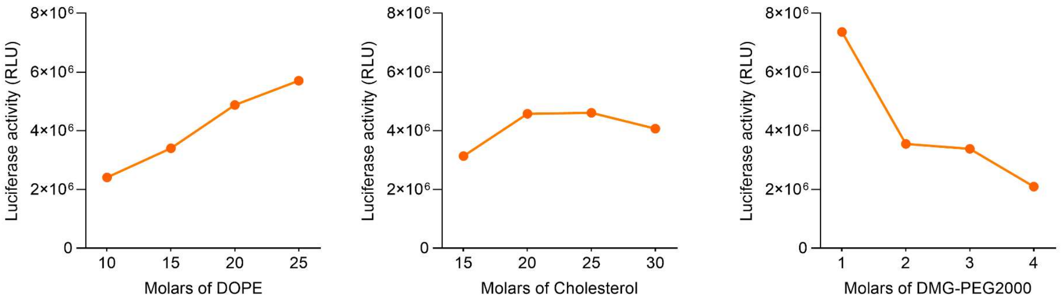

Supplementary Fig. 3 | The first-round orthogonal screening of the four components' molar ratios. The optimization of the formulation was based on four levels and the effect trend of each LNP component was shown.

a

Name	Factors (molar ratio)			
	6Ac1-C12	DOPE	Cholesterol	DMG-PEG2000
A-1	10	10	15	1
A-2	10	15	20	2
A-3	10	20	25	3
A-4	10	25	30	4
A-5	15	10	20	3
A-6	15	15	15	4
A-7	15	20	30	1
A-8	15	25	25	2
A-9	20	10	25	4
A-10	20	15	30	3
A-11	20	20	15	2
A-12	20	25	20	1
A-13	25	10	30	2
A-14	25	15	25	1
A-15	25	20	20	4
A-16	25	25	15	3

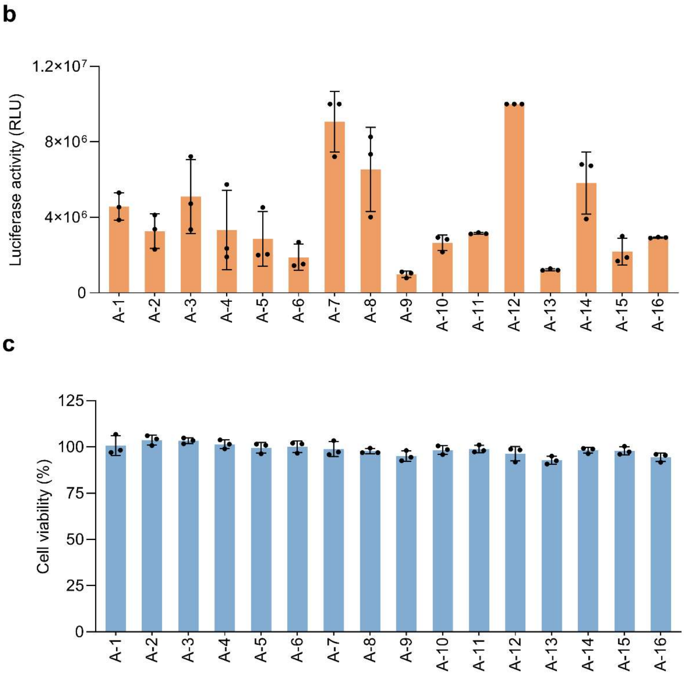

Supplementary Fig. 4 | Initial screening of component molar ratios in LNPs by orthogonal assay. a, Orthogonal array table L_{16} $(4)^4$ was used for screening and optimization of LNP formulations. **b**, Luciferase activity of the LNPs by different formulations. **c**, Cytotoxicity of nAcx-Cm LNPs in IGROV1 cells. Data are presented as mean ± s.d. (*n* = 3 biologically independent samples).

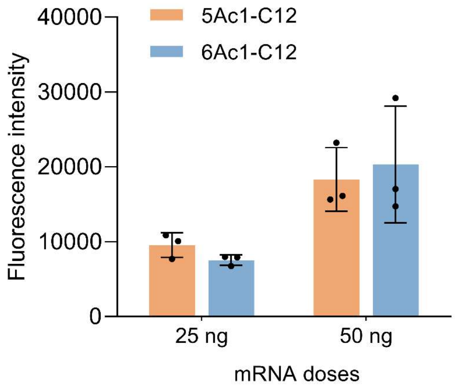

Supplementary Fig. 5 | In vitro mCherry mRNA delivery. The transfection was mediated by 5Ac1-C12 LNPs and 6Ac1-C12 LNPs at different mRNA dosages in IGROV1 cells. Data are presented as mean ± s.d. (*n* = 3 biologically independent samples).

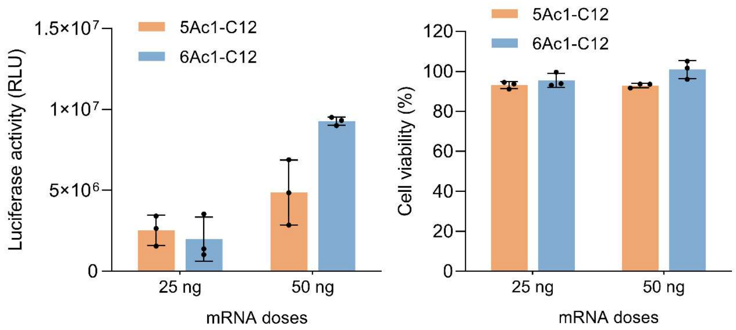

Supplementary Fig. 6 | In vitro Fluc mRNA delivery and cell viability of 5Ac1-C12 LNPs and 6Ac1-C12 LNPs. The IGROV1 cells were treated with 5Ac1-C12 LNPs and 6Ac-C12 LNPs encapsulating different dosages of mRNA (25 ng and 50 ng Fluc mRNA). Data are presented as mean ± s.d. (n = 3 biologically independent samples).

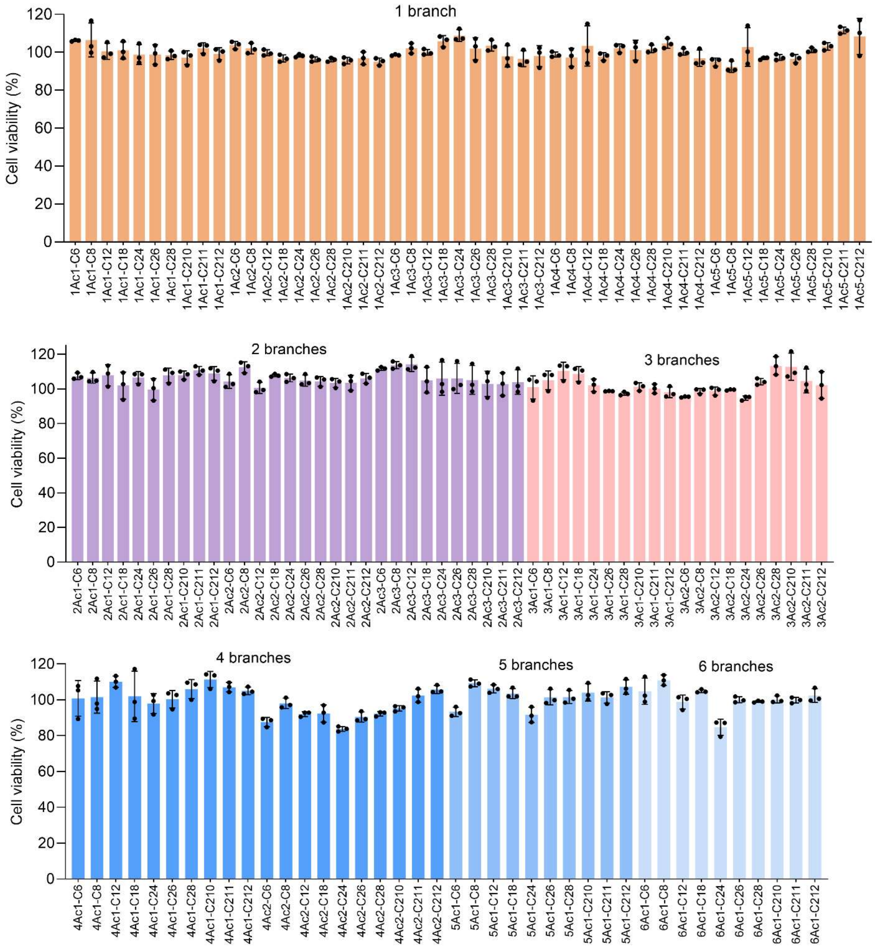

Supplementary Fig. 7 | Cytotoxicity analysis of LNP-mRNA formulations in IGROV1 cells. All nAcx-Cm LNPs in this library exhibited low cytotoxicity. Data are presented as mean ± s.d. (n = 3 biologically independent samples).

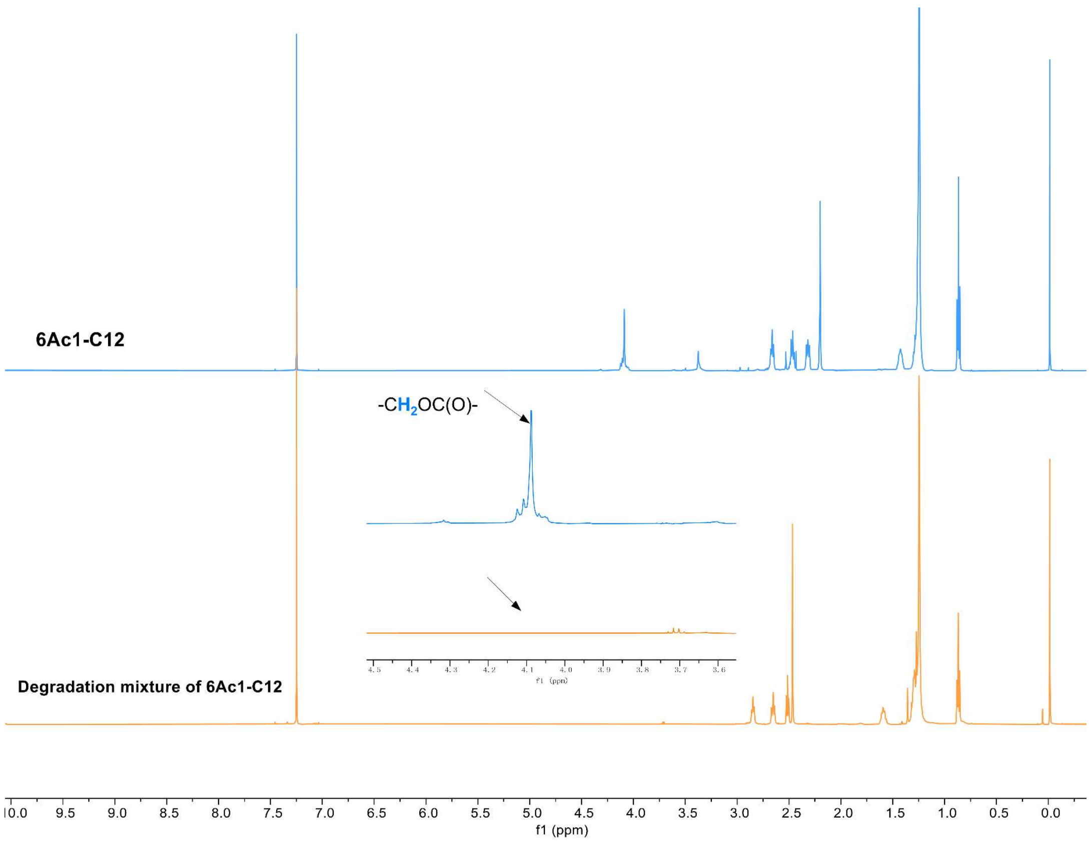

Supplementary Fig. 8 | ^{1}H NMR spectrum (CDCl₃) of 6Ac1-C12 lipid before and after degradation.

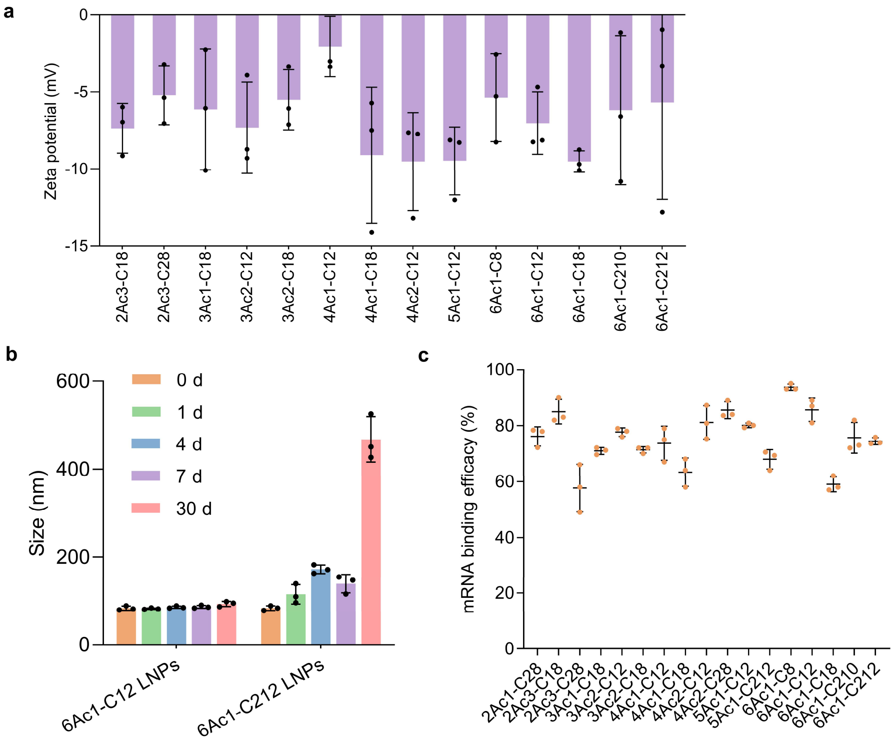

Supplementary Fig. 9 | Characterization of nAcx-Cm LNPs. Zeta potential (**a**), particle size (**b**) and mRNA binding efficacy (**c**) of representative candidates were evaluated. Particle sizes of 6Ac1-C12 LNPs remained unchanged post 30-day incubation. Data are presented as mean ± s.d. (n = 3 biologically independent samples).

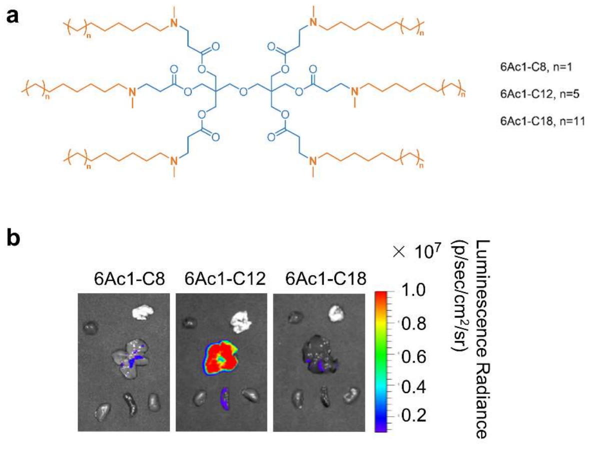

Supplementary Fig. 10 | Tail lengths of nAcx-Cm lipids affected the in vivo mRNA delivery efficacy. a, The structures of 6Ac1-C8, 6Ac1-C12 and 6Ac1-C18. **b**, C57BL/6 mice were treated with LNPs containing Fluc mRNA and bioluminescence was quantified 6 h post i.v. administration.

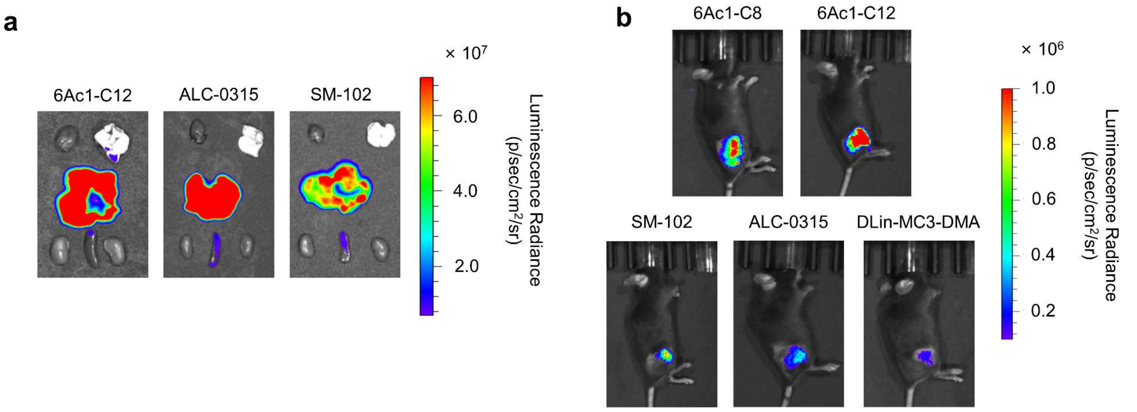

Supplementary Fig. 11 | The comparison of mRNA delivery efficiency of the optimal lipid 6Ac1-C12 with commercialized lipids by different administration routes. a, 6Ac1-C12 LNPs exhibited higher or comparable Fluc mRNA expression than SM-102 and ALC-0315 LNPs via i.v. injection. **b**, C57BL/6 mice were treated with LNPs containing Fluc mRNA and bioluminescence was quantified 6 h post intramuscular injection. 6Ac1-C12 LNPs outperformed SM-102, ALC-0315, and DLin-MC3-DMA LNPs by intramuscularly delivering mRNA.

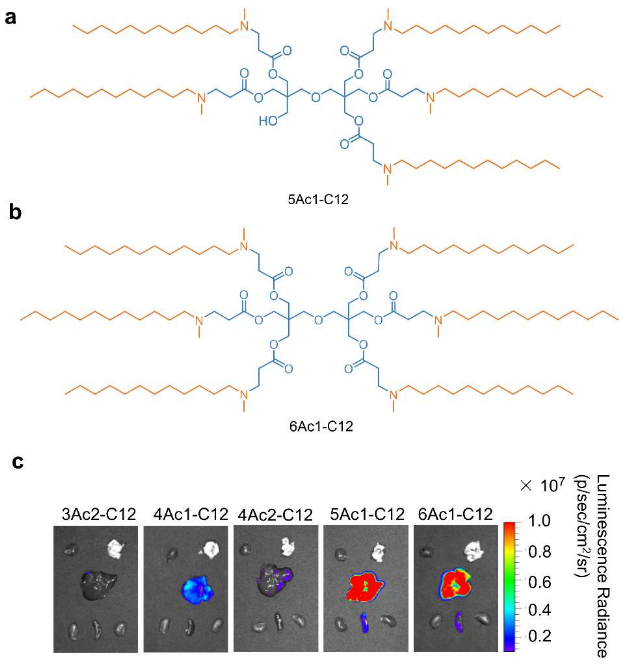

Supplementary Fig. 12 | Effect of different degradable cores on the mRNA delivery efficacy in vivo. a, Chemical structure of 5Ac1-C12. **b**, Structure of 6Ac1-C12. **c**, The bioluminescence imaging of individual organs was evaluated 6 h post injection.

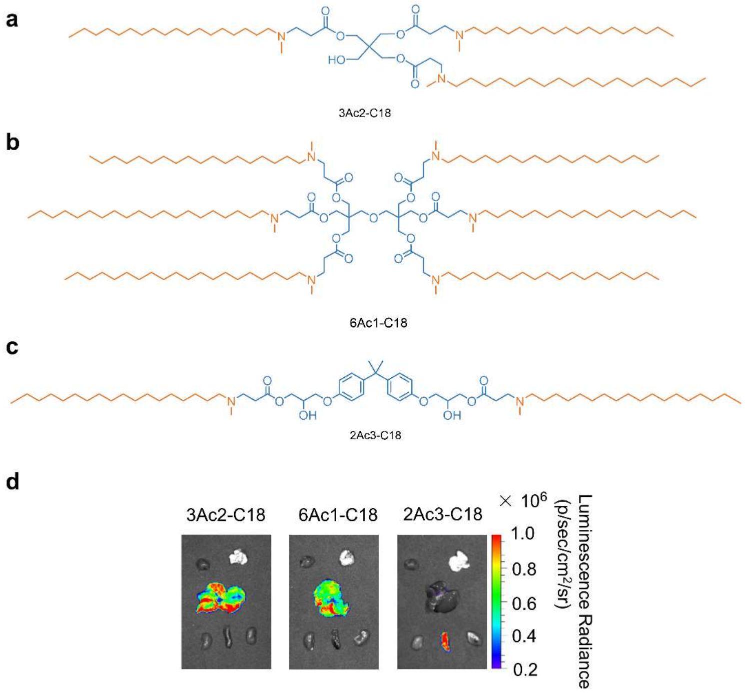

Supplementary Fig. 13 | Degradable cores affected the organ selectivity of mRNA delivery. **a**, Chemical structure of 3Ac2-C18. **b**, Structure of 6Ac1-C18. **c**, Structure of 2Ac3-C18. **d**, In vivo evaluation of 3Ac2-C18, 6Ac1-C18, and 2Ac3-C18 LNPs. 2Ac3-C18 LNPs exhibited spleen-specific mRNA expression in vivo.

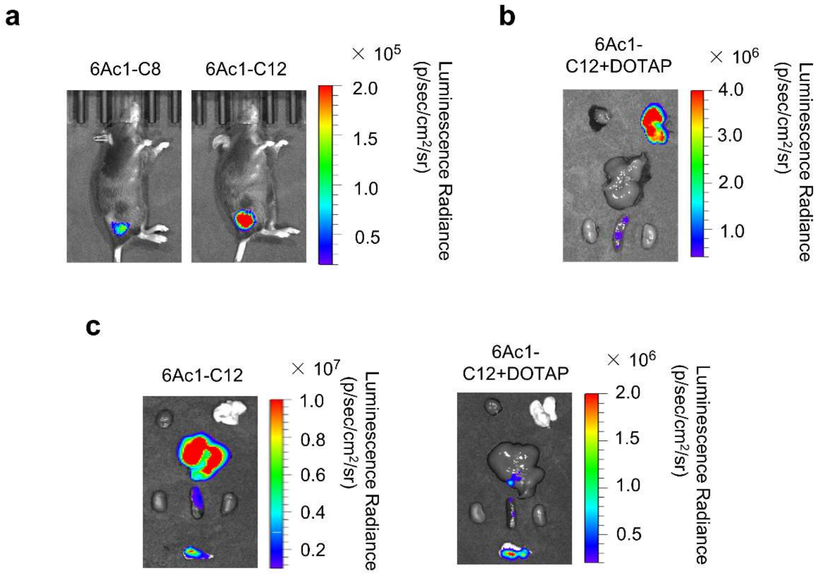

Supplementary Fig. 14 | Administration routes influenced the organ-targeting and mRNA delivery efficacy. **a**, 6Ac1-C12 and 6Ac1-C8 LNPs enabled Fluc mRNA expression via subcutaneous injection. **b**, Lung-specific mRNA expression could be achieved by i.v. injection of 6Ac1-C12 LNPs incorporating the 50% SORT molecule DOTAP. **c**, 6Ac1-C12 LNPs enabled mRNA delivery to the pancreas post intraperitoneal administration. 50% DOTAP incorporation into 6Ac1-C12 LNPs mediated pancreas specific mRNA expression after intraperitoneal administration.

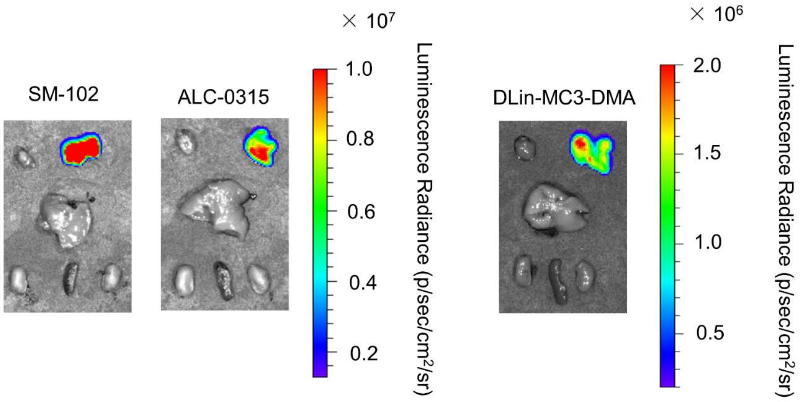

Supplementary Fig. 15 | The 3-Comp Lung strategy exhibited universal applicability to other cationic lipids for lung-selective mRNA expression. SM-102, ALC-0315, and DLin-MC3-DMA based 3-Comp LNPs mediated mRNA expression in the lung.

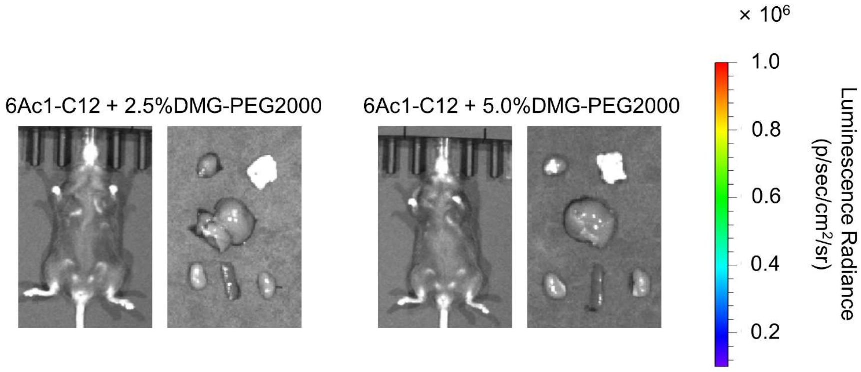

Supplementary Fig. 16 | In vivo quantification of Fluc mRNA delivery via 6Ac1-C12/DMG-PEG2000 two component LNPs. 2.5% or 5% (wt) DMG-PEG2000 was used here. The two component LNPs could not enable efficacious mRNA delivery in vivo.

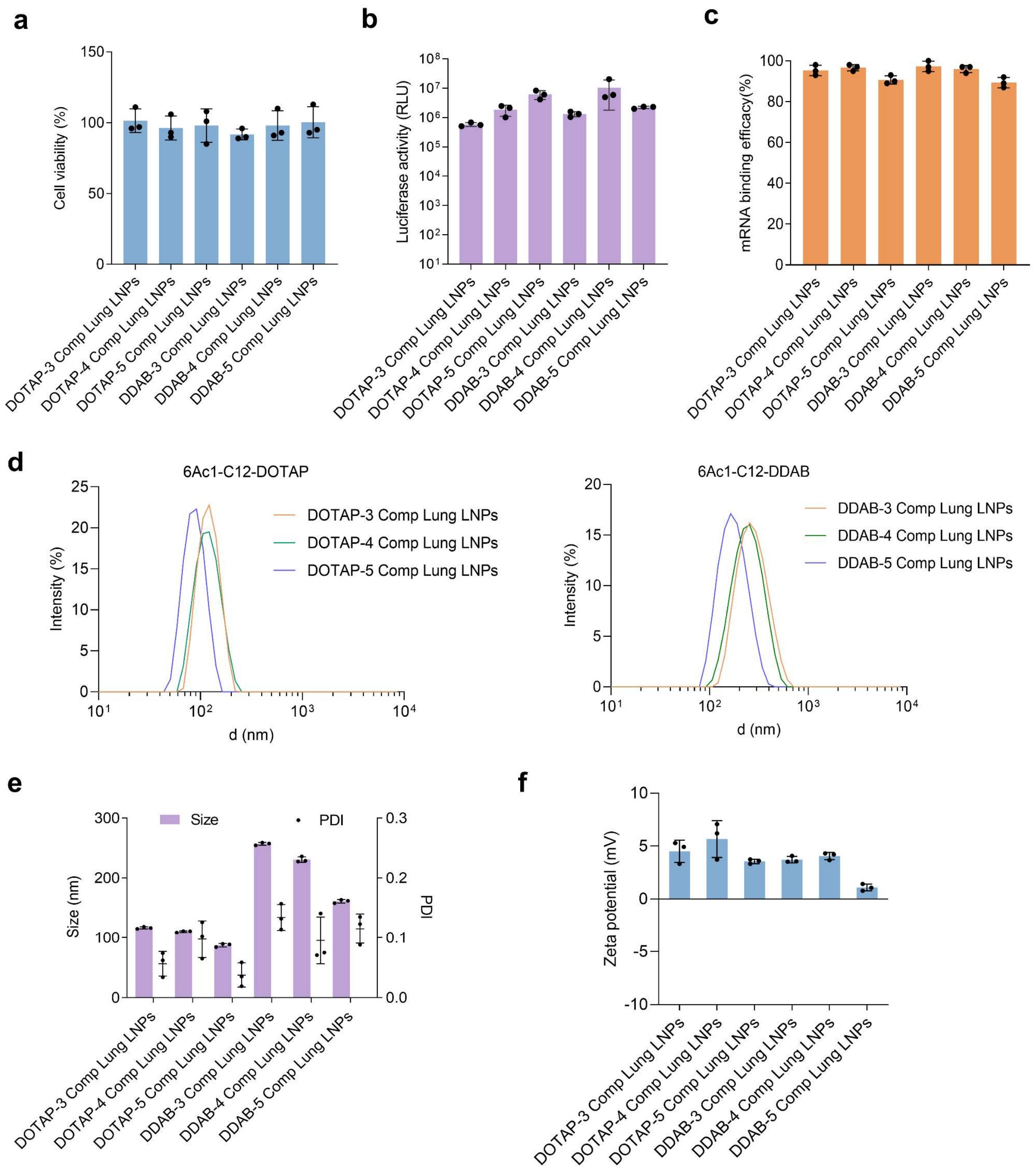

Supplementary Fig. 17 | Characterization of 6Ac1-C12 Lung LNPs with different components. Cell viability (**a**), in vitro mRNA delivery efficacy (**b**), mRNA binding (**c**), particle size (**d**), PDI (**e**), and zeta potential (**f**) were evaluated. Data are presented as mean ± s.d. (n = 3 biologically independent samples).

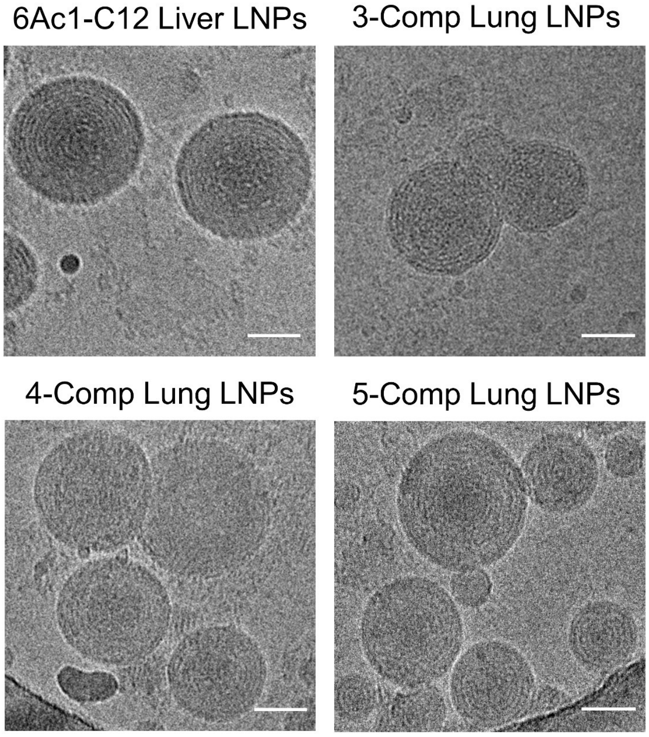

Supplementary Fig. 18 | Representative cryo-transmission electron microscope (cryo-TEM) images of different nAcx-Cm LNPs. All nAcx-Cm Liver and Lung LNPs presented a regular spherical morphology. Scale bar, 50 nm.

Levels	Factors (molar ratio)			
	6Ac1-C12	DDAB	Cholesterol	DMG-PEG 2000
1	40	20	20	0.2
2	50	30	30	0.4
3	60	40	40	0.8
4	70	50	50	1.2

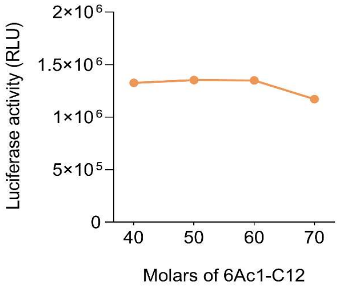

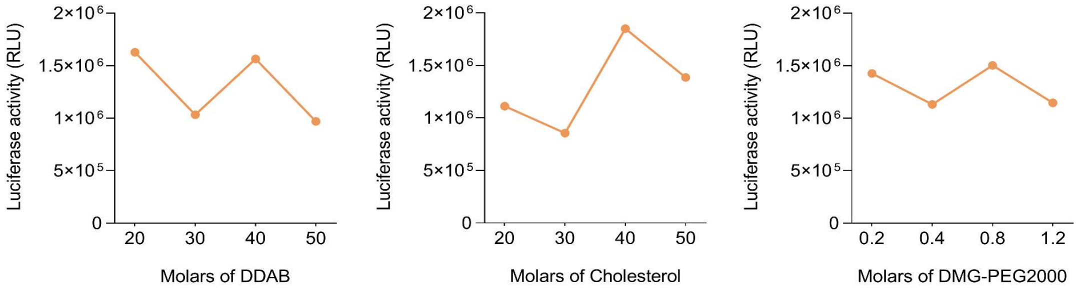

Supplementary Fig. 19 | Screening and optimization of 4-Comp Lung LNPs by orthogonal assay. Data showed four components and their effect on the luminescence intensity.

	Factors (molar ratio)			
	6Ac1-C12	DDAB	Cholesterol	DMG-PEG 2000
1	40	20	20	0.2
2	40	30	30	0.4
3	40	40	40	0.8
4	40	50	50	1.2
5	50	20	30	0.8
6	50	30	20	1.2
7	50	40	50	0.2
8	50	50	40	0.4
9	60	20	40	1.2
10	60	30	50	0.8
11	60	40	20	0.4
12	60	50	30	0.2
13	70	20	50	0.4
14	70	30	40	0.2
15	70	40	30	1.2
16	70	50	20	0.8

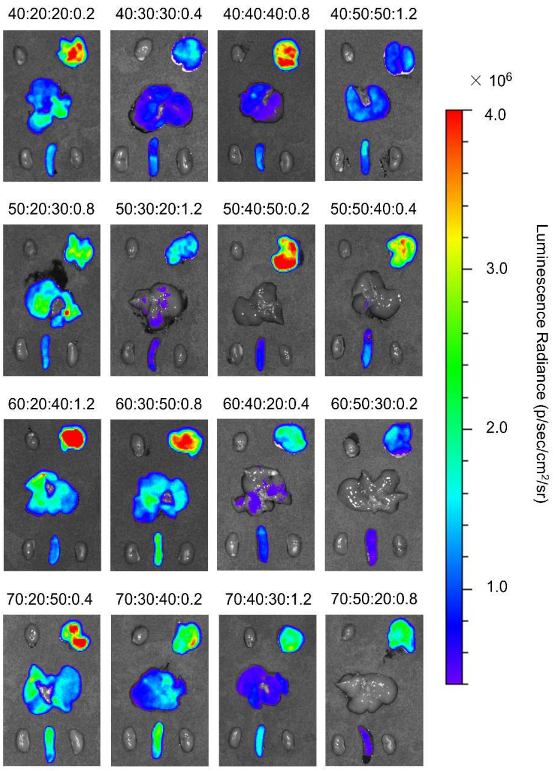

Supplementary Fig. 20 | The 16 4-Comp Lung LNP formulations were evaluated in vivo by orthogonal methods. DDAB based 4-Comp Lung LNPs exhibited superior delivery efficiency and lung targeting ability at the 6Ac1-C12/DDAB/cholesterol/DMG-PEG2000 molar ratio of 50/40/50/0.2.

a

	Factors (molar ratio)			
	6Ac1-C12	DDAB	Cholesterol	DMG-PEG 2000
1	60	20	40	1.2
2	60	30	40	1.2
3	60	40	40	1.2
4	60	50	40	1.2
5	60	60	40	1.2
6	60	70	40	1.2
7	60	80	40	1.2
8	50	40	50	0.2
9	50	50	50	0.2
10	50	60	50	0.2
11	50	70	50	0.2

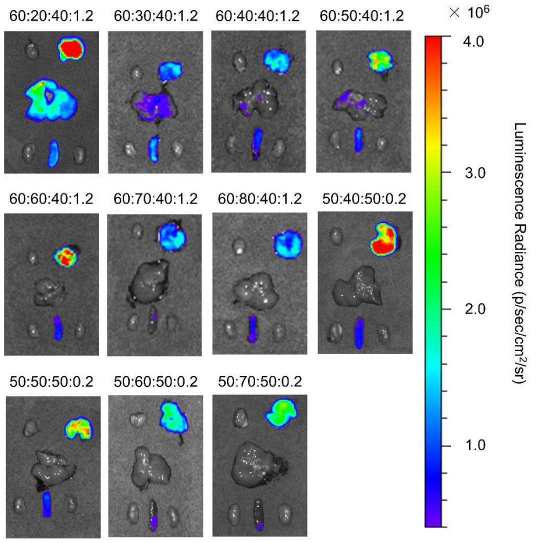

b

	Factors (molar ratio)			
	6Ac1-C12	DOTAP	Cholesterol	DMG-PEG 2000
1	60	60	40	1.2
2	50	40	50	0.2
3	50	70	50	0.2

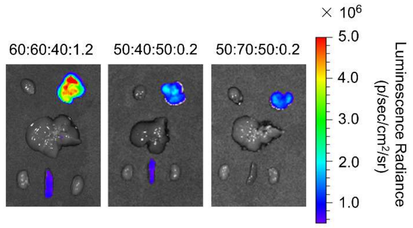

Supplementary Fig. 21 | The second round of 4-Comp Lung LNP formulation screening was evaluated by orthogonal methods. a, Effect of permanently cationic lipid DDAB content on mRNA targeted delivery. **b**, DOTAP based 4-Comp Lung LNPs exhibited superior delivery efficiency and lung targeting ability than DDAB 4-Comp counterparts at the 6Ac1-C12/DOTAP/cholesterol/DMG-PEG2000 molar ratio of 60/60/40/1.2.

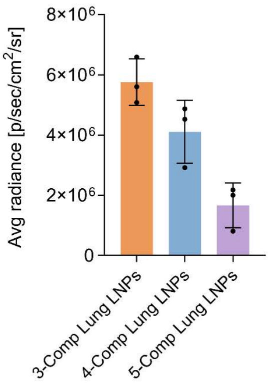

Supplementary Fig. 22 | In vivo quantification of Fluc mRNA delivery to the lung by DOTAP based Lung LNPs. The 3-component LNPs outperformed their 4- and 5-component counterparts in Fluc mRNA delivery efficiency. Data are presented as mean ± s.d. (n = 3 biologically independent mice).

a

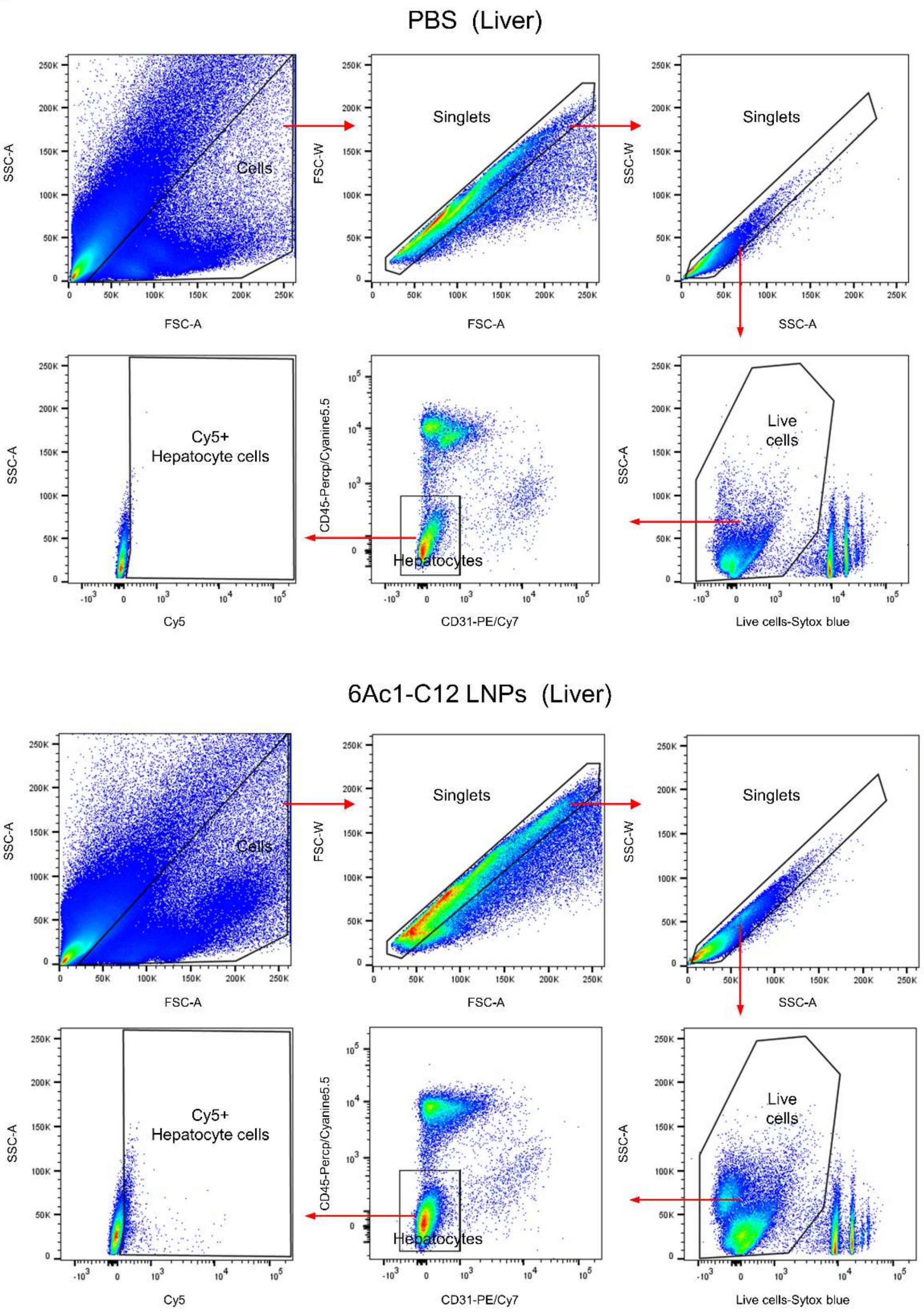
PBS (Liver)
Cells
Singlets
Singlets
SSC-A
FSC-A
FSC-W
SSC-W
Cy5+
Hepatocyte cells
Hepatocytes
Live
cells
CD45-Percp/Cyanine5.5
Cy5
CD31-PE/Cy7
Live cells-Sytox blue
6Ac1-C12 LNPs (Liver)
Cells
Singlets
Singlets
Cy5+
Hepatocyte cells
Hepatocytes
Live
cells

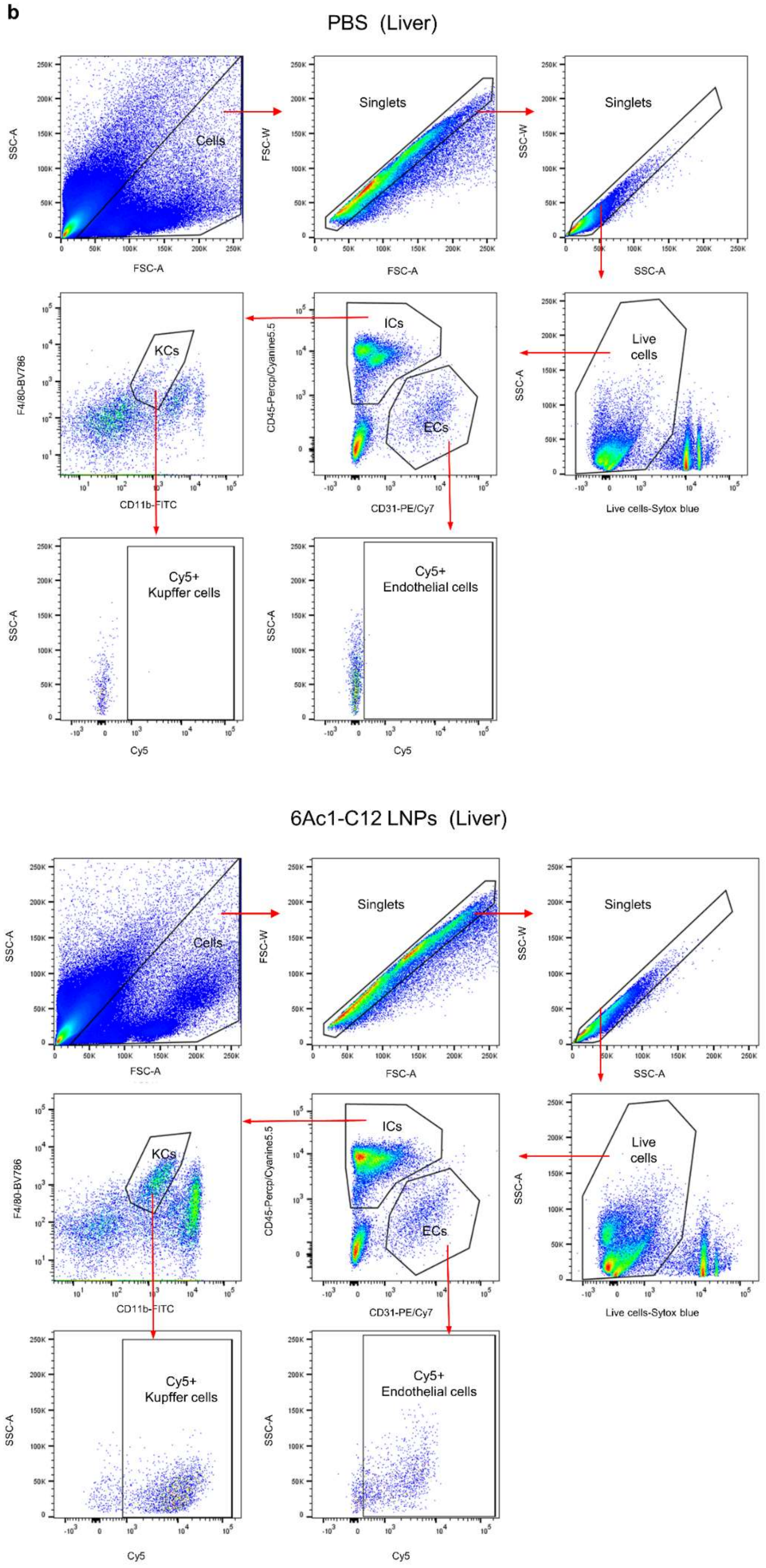
b
PBS (Liver)
SSC-A
FSC-A
Cells
FSC-W
Singlets
SSC-W
F4/80-BV786
CD11b-FITC
KCs
CD45-Percp/Cyanine5.5
CD31-PE/Cy7
ICs
ECs
Live cells
Live cells-Sytox blue
Cy5
Cy5+
Kupffer cells
Cy5+
Endothelial cells
6Ac1-C12 LNPs (Liver)

Supplementary Fig. 23 | The FACS gating strategy for analysis of LNP-mRNA (Cy5-mRNA) distribution in liver cells. a, Gating strategy for hepatocyte cells. **b**, Gating strategy for liver non-parenchymal cells. Sytox^{TM} Blue was used to define live cells. CD45- and CD31- were used to define hepatocyte cells. CD45+ and CD31- were used to define immune cells, and CD45- and CD31+ were used to define endothelial cells. CD11b+ and F4/80+ were used to define kupffer cells. Gates for Cy5+ in cell types were drawn based on the PBS injected control mice. C57BL/6 mice were i.v. injected with 6Ac1-C12 Liver LNPs (Cy5-mRNA, 0.5 mg kg^{-1}) and Cy5+ in given cell types was detected by flow cytometry.

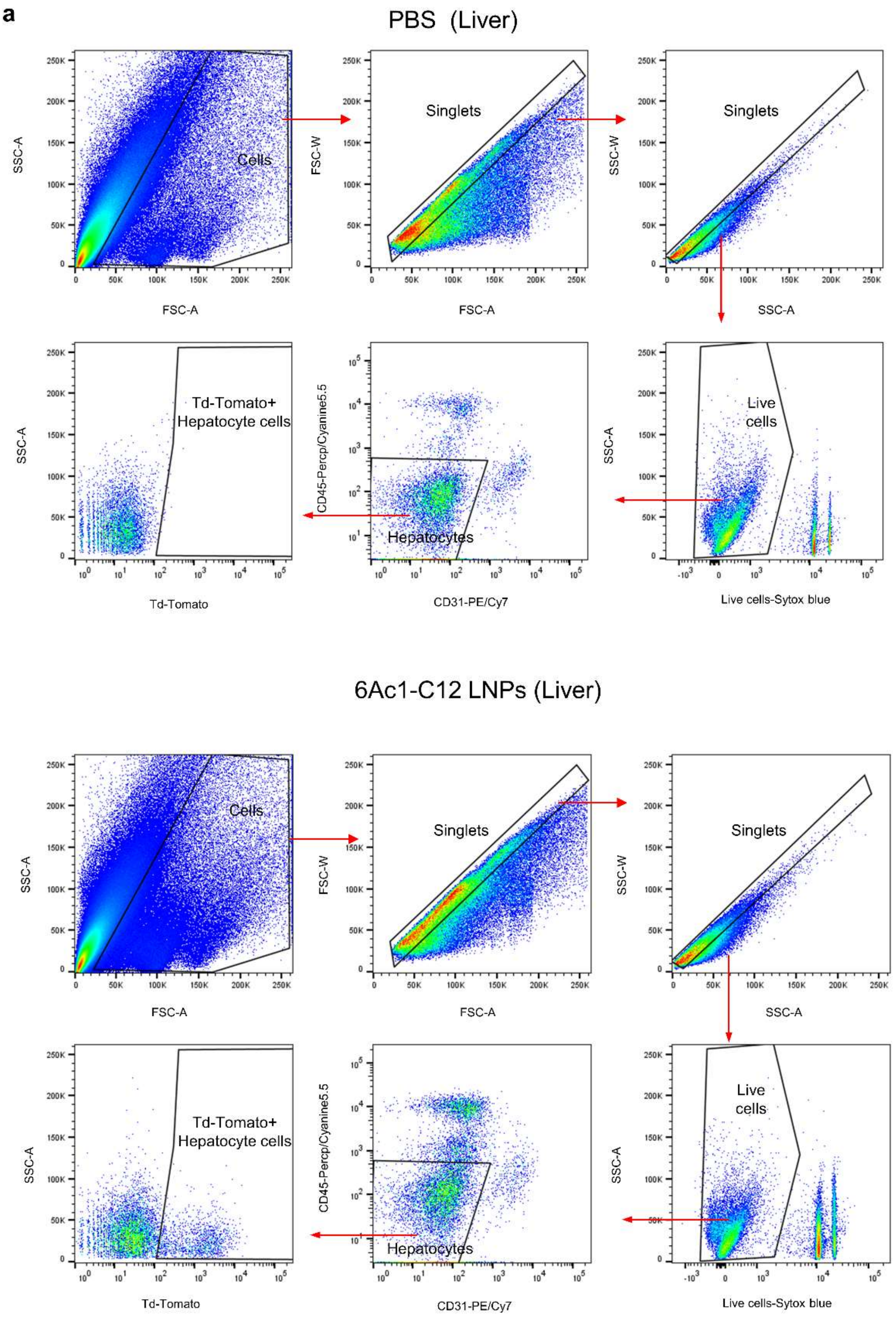
a
PBS (Liver)
Cells
SSC-A
FSC-A
Singlets
FSC-W
FSC-A
Singlets
SSC-W
SSC-A
Live cells
SSC-A
Live cells-Sytox blue
Hepatocytes
CD45-Percp/Cyanine5.5
CD31-PE/Cy7
Td-Tomato+ Hepatocyte cells
SSC-A
Td-Tomato
6Ac1-C12 LNPs (Liver)
Cells
SSC-A
FSC-A
Singlets
FSC-W
FSC-A
Singlets
SSC-W
SSC-A
Live cells
SSC-A
Live cells-Sytox blue
Hepatocytes
CD45-Percp/Cyanine5.5
CD31-PE/Cy7
Td-Tomato+ Hepatocyte cells
SSC-A
Td-Tomato

b

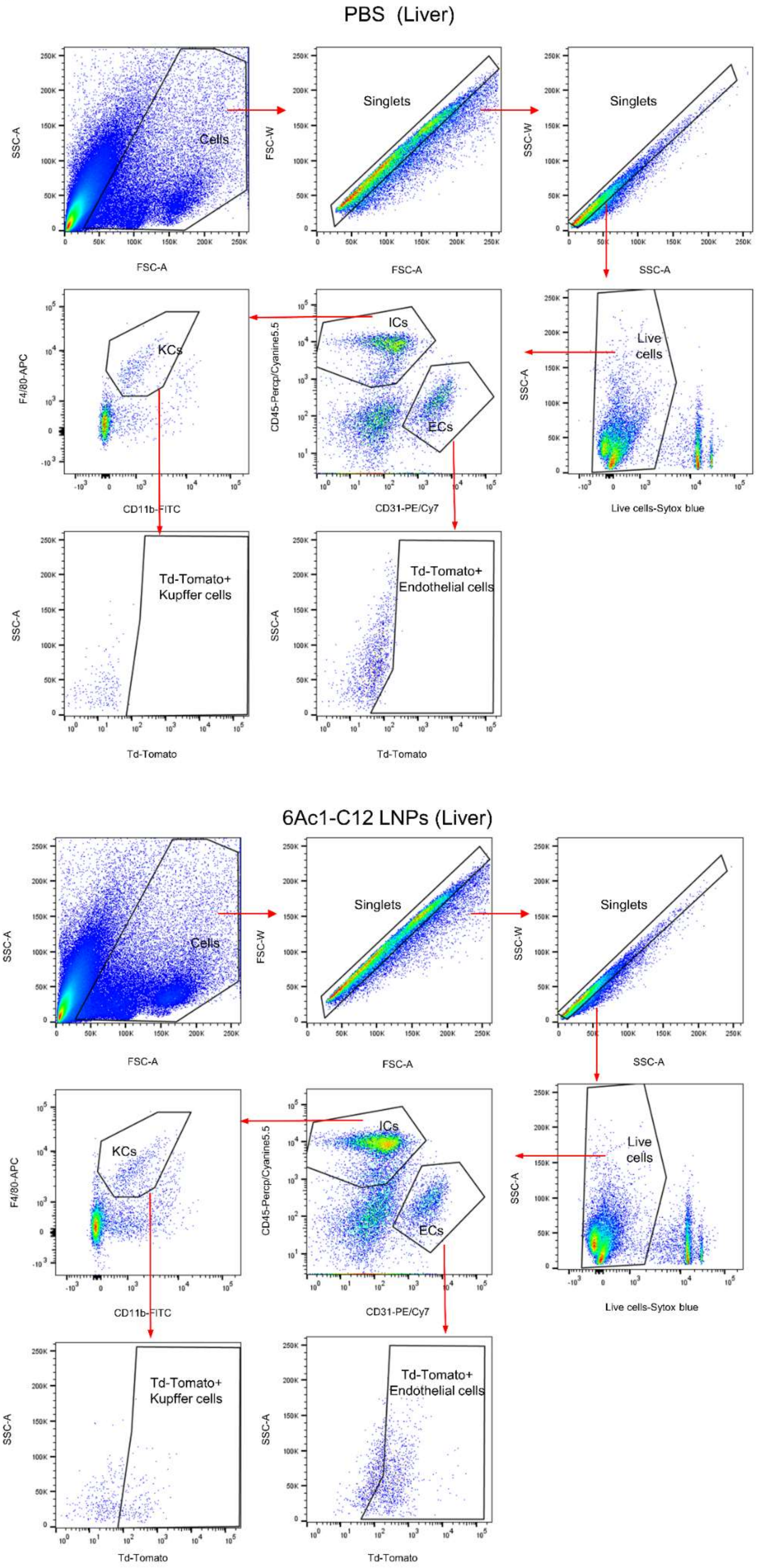
PBS (Liver)
Cells
Singlets
Singlets
SSC-A
FSC-A
FSC-W
SSC-W
KCs
ICs
ECs
Live cells
F4/80-APC
CD11b-FITC
CD45-Percp/Cyanine5.5
CD31-PE/Cy7
Live cells-Sytox blue
Td-Tomato+ Kupffer cells
Td-Tomato+ Endothelial cells
Td-Tomato
6Ac1-C12 LNPs (Liver)

Supplementary Fig. 24 | The FACS gating strategy for analysis of tdTomato expression in liver cells. a, Gating strategy for hepatocyte cells. **b**, Gating strategy for liver non-parenchymal cells. Sytox^{TM} Blue was used to identify live and dead cells. CD45- and CD31- were used to define hepatocyte cells. CD45+ and CD31- were used to define immune cells, and CD45- and CD31+ were used to define endothelial cells. CD11b+ and F4/80+ were used to define kupffer cells. Gates for tdTomato+ in cell types were drawn based on the PBS injected control mice. Ai9 mice were i.v. injected with 6Ac1-C12 Liver LNPs (Cre mRNA, 0.25 mg kg^{-1}) and tdTomato+ in given cell types was detected by flow cytometry.

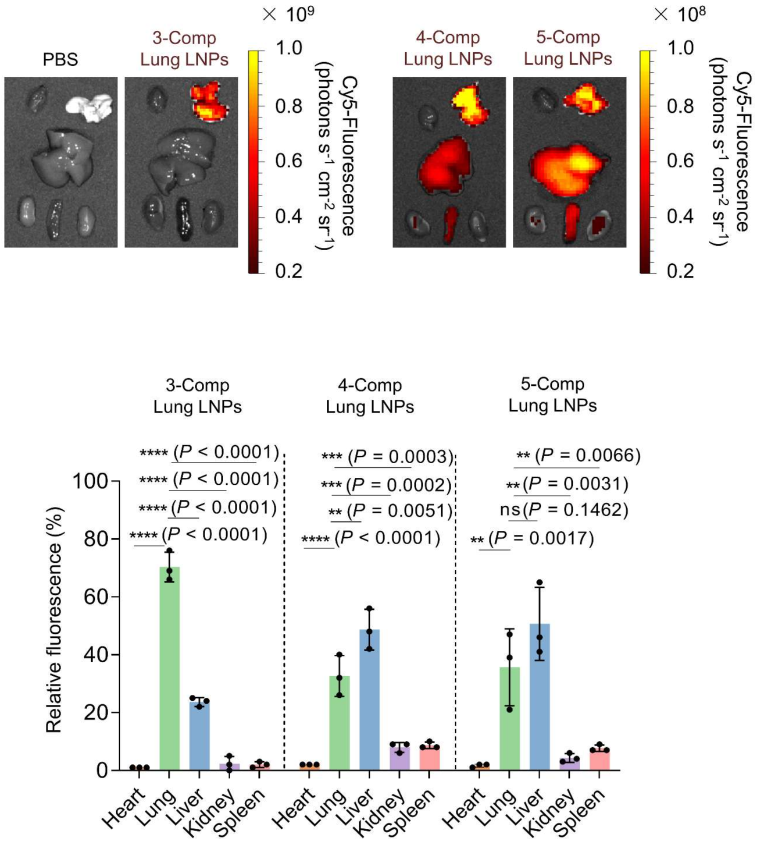

Supplementary Fig. 25 | Organ distribution and quantification were determined by 6Ac1-C12 LNPs loading Cy5-mRNA. C57BL/6 mice were i.v. treated by LNPs with different components at a Cy5-mRNA dose of 0.5 mg kg^{-1}. 3-Comp Lung LNPs accumulated specifically in the lung. Accumulation percentage (%) was calculated by total radiance subtracting that of PBS group. All statistical significances were calculated using one-way ANOVA with Dunnett's multiple comparisons test: $^{****}P < 0.0001$; $^{***}P < 0.001$; $^{**}P < 0.01$; $^{*}P < 0.05$; ns, no significant difference. All data are from n = 3 biologically independent mice.

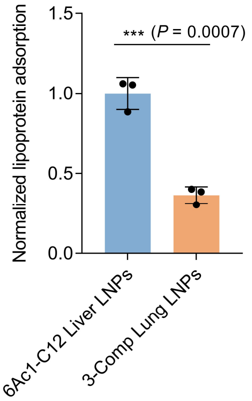

Supplementary Fig. 26 | Analysis of LNP protein coronas. 3-Comp Lung LNPs exhibited decreased lipoprotein adsorption compared to the typical Liver LNPs. Data are presented as mean ± s.d. (n = 3 biologically independent samples). Statistical significance was calculated with a two-tailed unpaired t-test: $^{****}P < 0.0001$; $^{***}P < 0.001$; $^{**}P < 0.01$; $^{*}P < 0.05$.

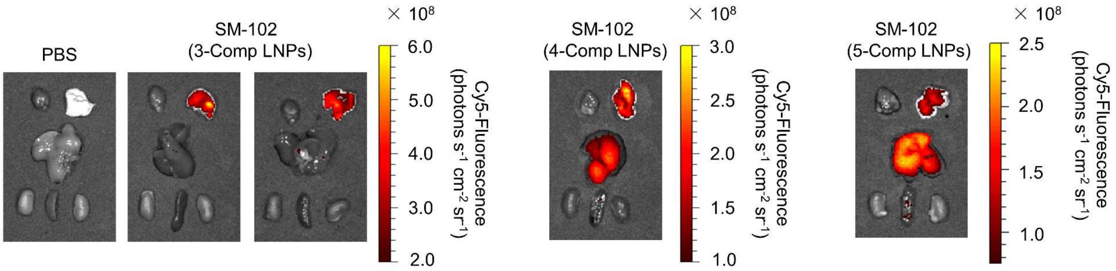

Supplementary Fig. 27 | The 3-Comp Lung strategy exhibited universal applicability to other cationic lipids for lung-targeted accumulation. C57BL/6 mice were i.v. treated with SM-102 3-, 4-, and 5-Comp Lung LNPs at the Cy5-mRNA dosage of 0.5 mg kg^{-1}. The 3-component strategy outperformed their 4- and 5-component counterparts in targeted accumulation.

PBS (Lung)

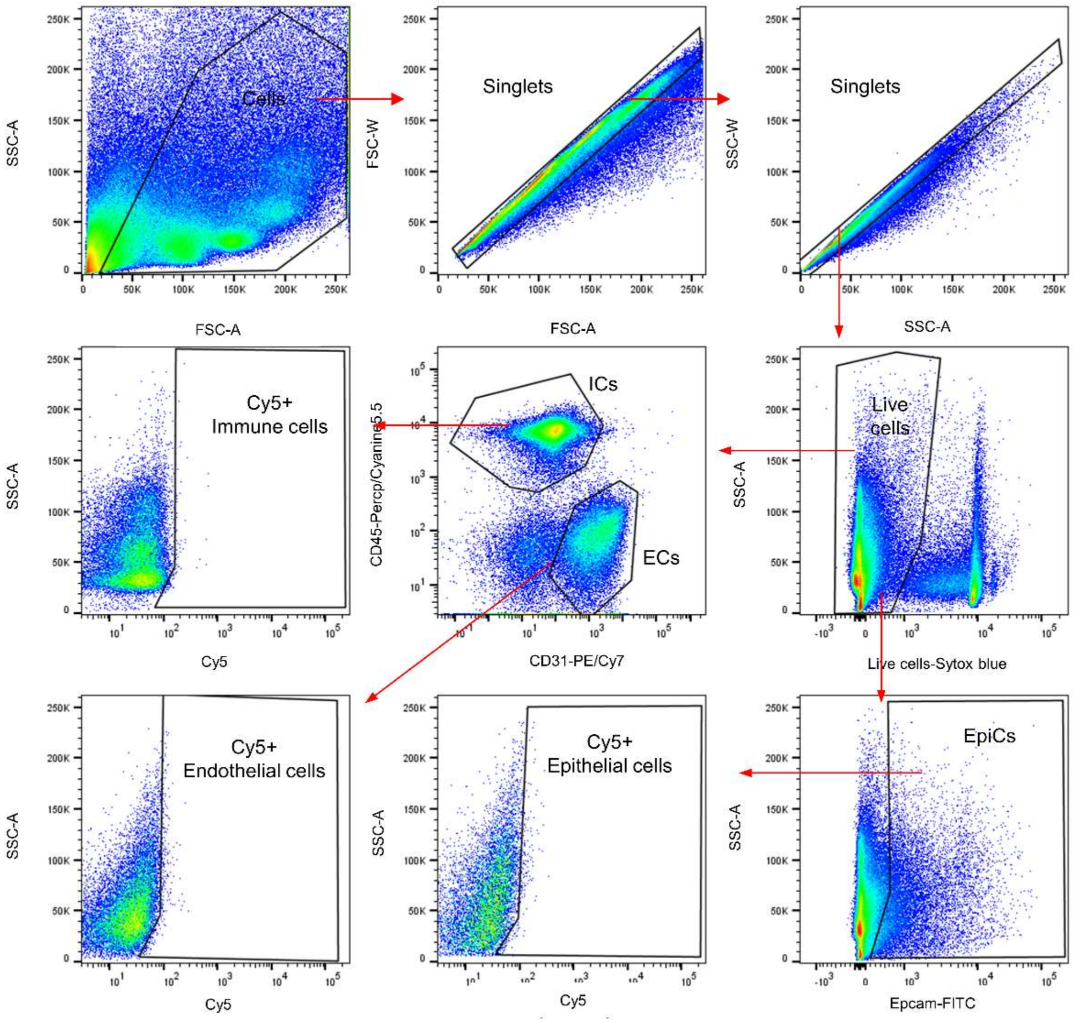
Cells
Singlets
Singlets
SSC-A
FSC-A
FSC-W
SSC-W
Cy5+
Immune cells
ICs
ECs
Live
cells
CD45-Percp/Cyanine5.5
CD31-PE/Cy7
Live cells-Sytox blue
Cy5
Cy5+
Endothelial cells
Cy5+
Epithelial cells
EpiCs
Epcam-FITC

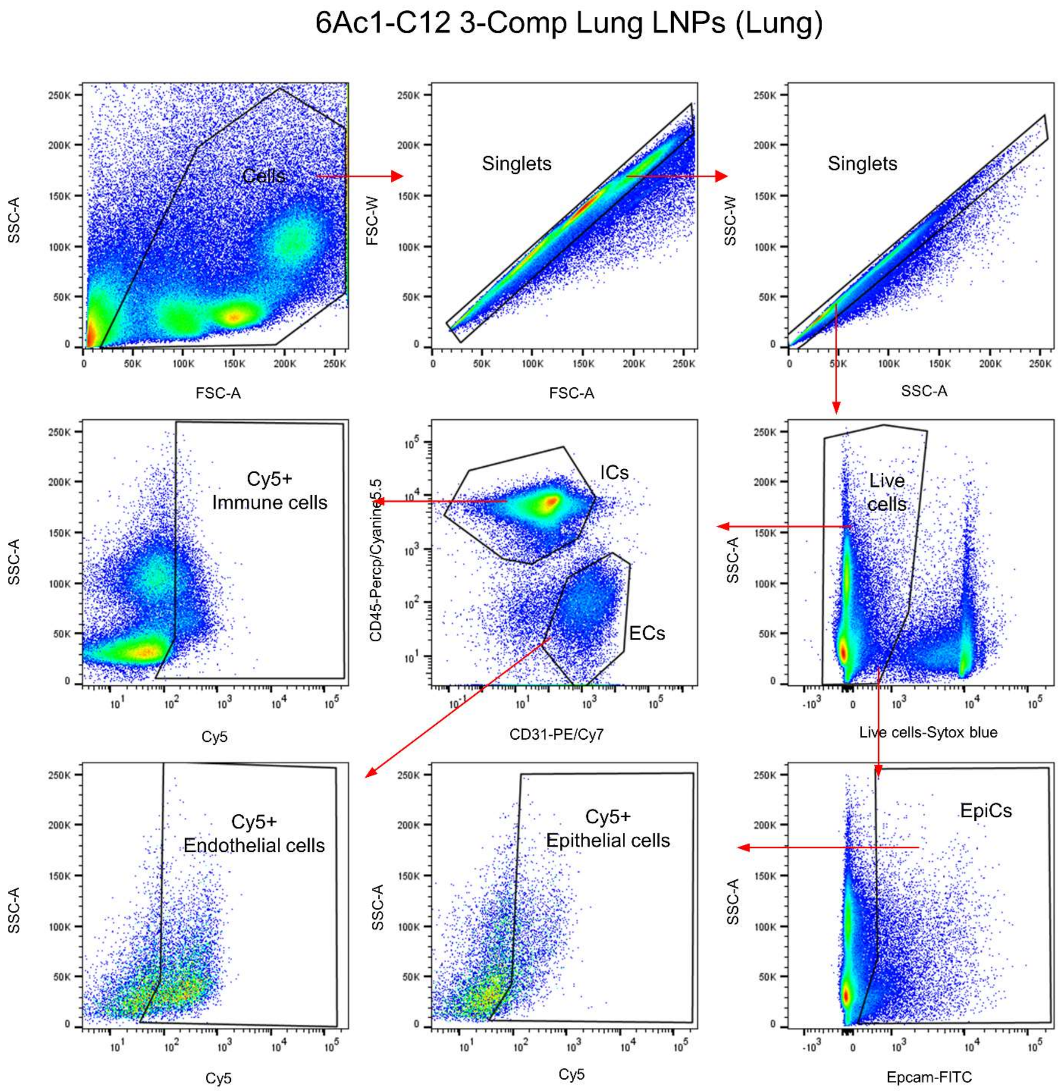

Supplementary Fig. 28 | The FACS gating strategy for analysis of LNP-mRNA (Cy5-mRNA) accumulation in lung cells. Sytox^{TM} Blue was used to define live and dead cells. EpCam+ was utilized to define epithelial cells, CD45+ and CD31- was used to define immune cells, and CD45- and CD31+ were used to define endothelial cells. Gates for Cy5+ in cell types were drawn based on the PBS injected control mice. C57BL/6 mice were i.v. injected with 6Ac1-C12 3-Comp Lung LNPs (Cy5-mRNA, 0.5 mg kg^{-1}) and Cy5+ in given cell types was detected by flow cytometry.

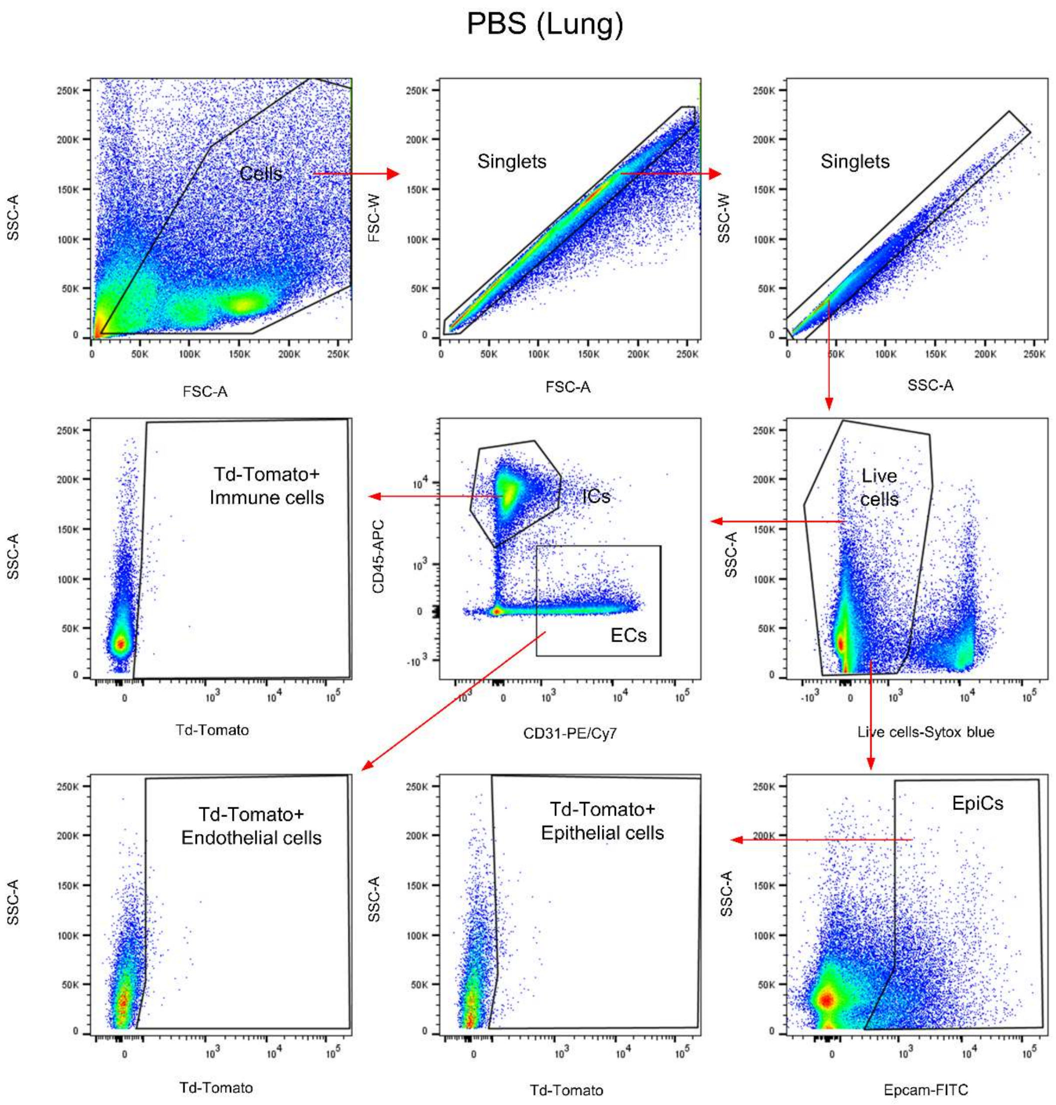
PBS (Lung)
Cells
Singlets
Singlets
SSC-A
FSC-A
FSC-W
FSC-A
SSC-W
SSC-A
Td-Tomato+
Immune cells
ICs
ECs
Live
cells
Td-Tomato
CD45-APC
CD31-PE/Cy7
Live cells-Sytox blue
Td-Tomato+
Endothelial cells
Td-Tomato+
Epithelial cells
EpiCs
Td-Tomato
Td-Tomato
Epcam-FITC

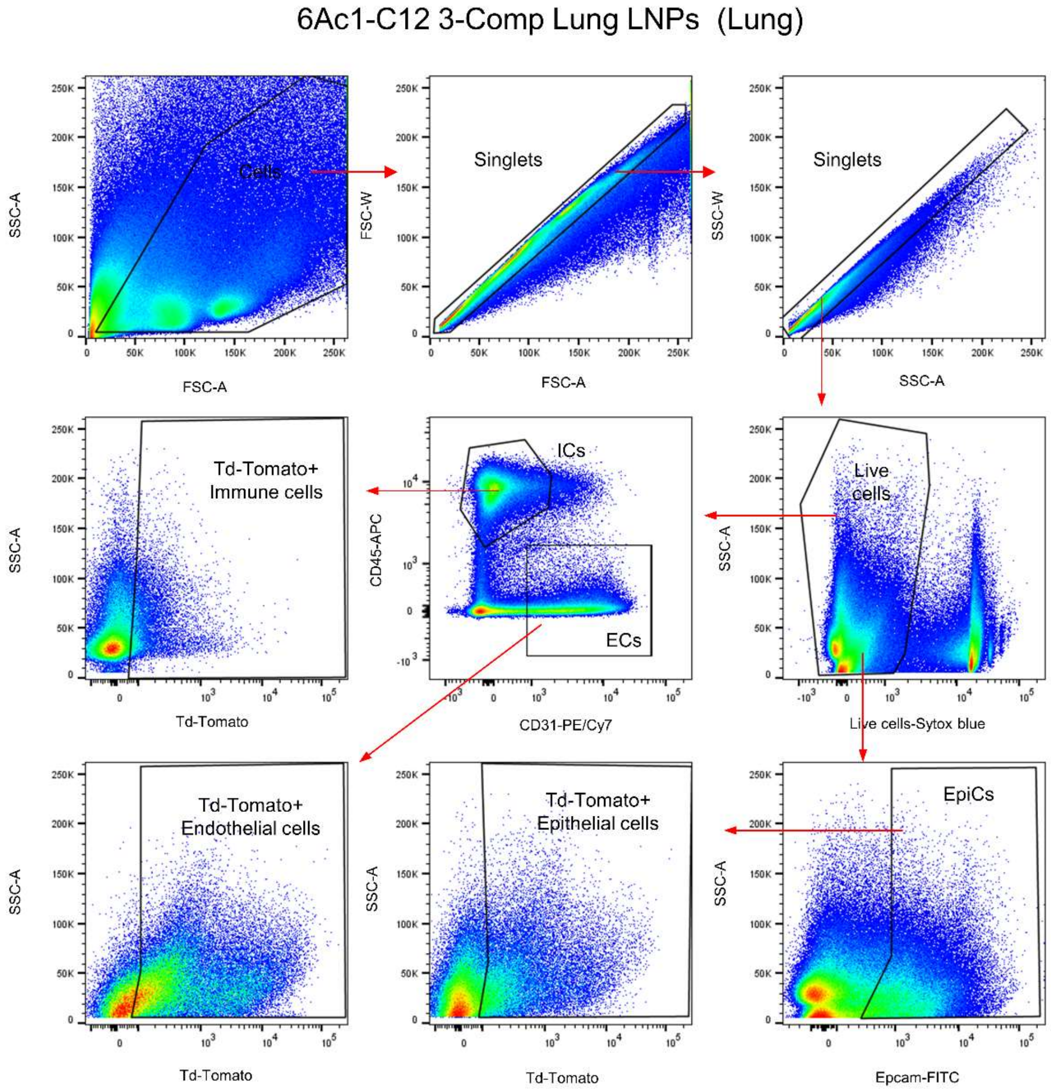

Supplementary Fig. 29 | The FACS gating strategy for analysis of tdTomato expression in lung cells. Sytox™ Blue was used to identify live and dead cells. EpCam+ was utilized to define epithelial cells, CD45+ and CD31- was used to define immune cells, and CD45- and CD31+ were used to define endothelial cells. Gates for tdTomato+ in cell types were drawn based on the PBS injected control mice. Ai9 mice were i.v. injected with 6Ac1-C12 3-Comp Lung LNPs (Cre mRNA, 0.25 mg kg^{-1}) and tdTomato+ in given cell types was detected by flow cytometry.

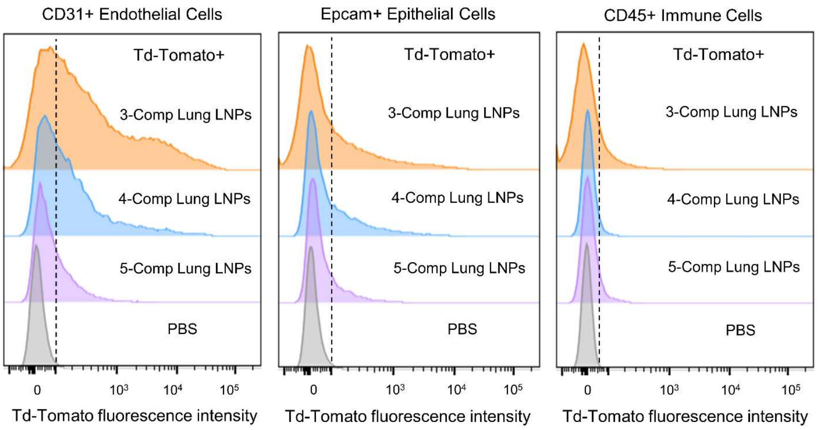

Supplementary Fig. 30 | FACS revealed the mRNA translation in different pulmonary cell subsets after the treatment of Lung LNPs.

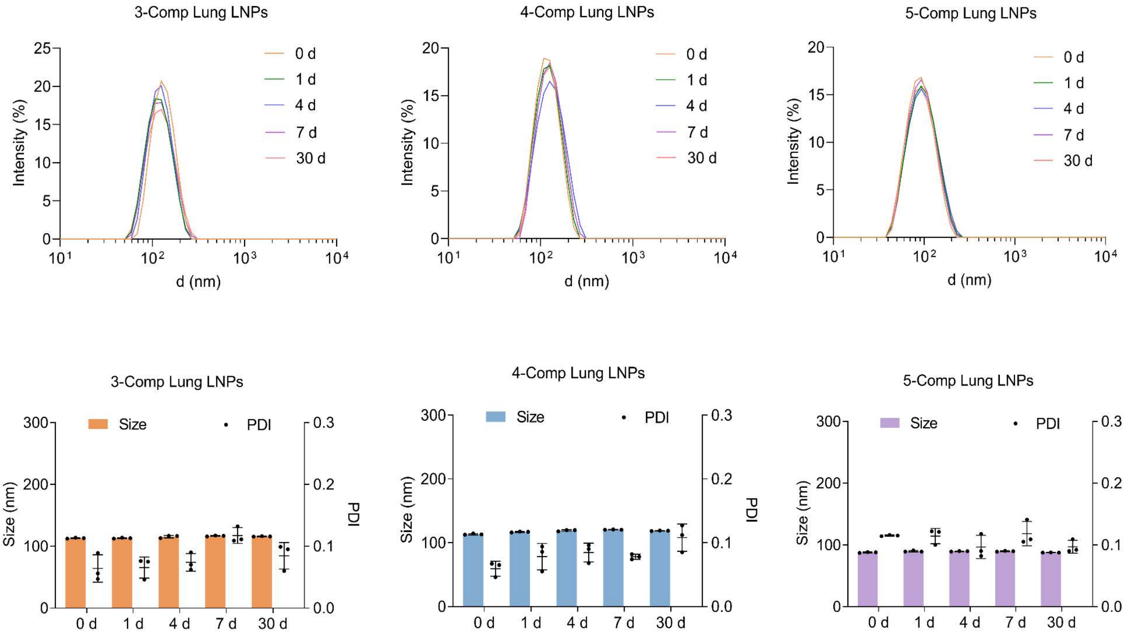

Supplementary Fig. 31 | Stability of 6Ac1-C12 Lung LNPs with different components. DOTAP based 6Ac1-C12 Lung LNPs all remained stable post 30-d incubation. Data are presented as mean ± s.d. (*n* = 3 biologically independent samples).

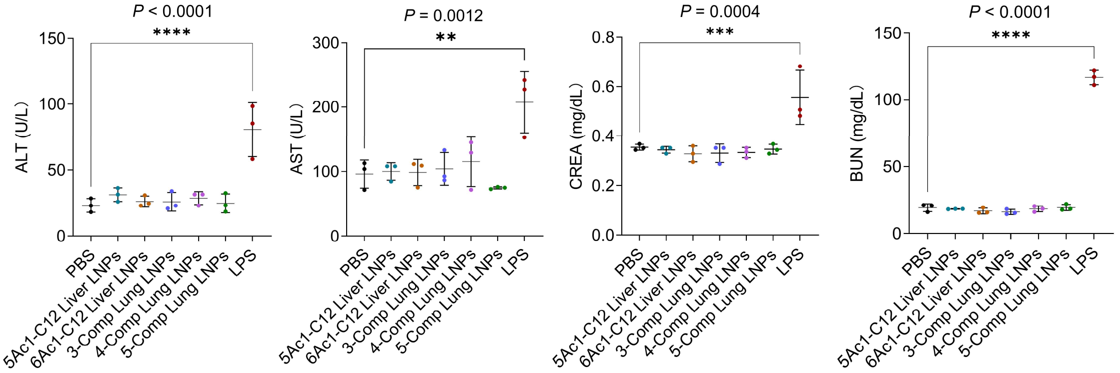

Supplementary Fig. 32 | nAcx-Cm Liver and Lung LNPs were well tolerated in vivo. nAcx-Cm Liver and Lung LNPs were i.v. administrated to C57BL/6 mice. Lipopolysaccharide (LPS, 5 mg kg^{-1}, intraperitoneal) was used as the positive control and PBS (i.v.) was examined as the negative control. Liver function (ALT and AST) and kidney function (BUN and CREA) and were evaluated. LPS treated mice showed severe kidney and liver injury. There was no significant difference between the nAcx-Cm LNPs and PBS groups. All statistical significances were calculated using one-way ANOVA with Dunnett's multiple comparisons test: $^{****}P < 0.0001$; $^{***}P < 0.001$; $^{**}P < 0.01$; $^{*}P < 0.05$. All data are from $n = 3$ biologically independent mice.

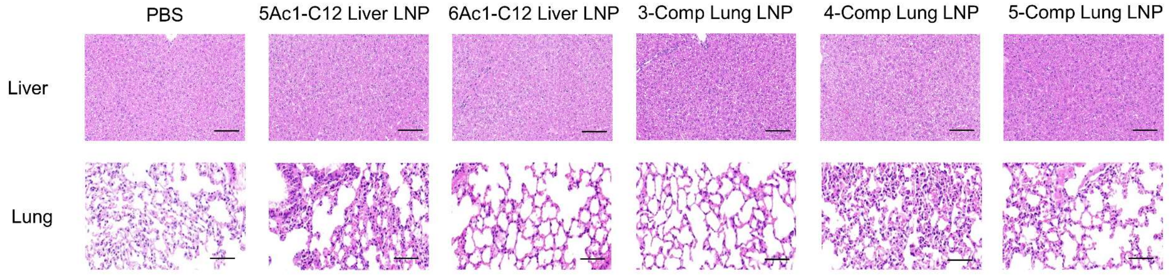

Supplementary Fig. 33 | Liver and lung specific nAcx-Cm LNPs were well tolerated in vivo. 5Ac1-C12 and 6Ac1-C12 Liver LNPs (liver specific) and 3-, 4-, and 5-Comp Lung LNPs (lung specific) were i.v. administrated to C57BL/6 mice. PBS (i.v.) was examined as the negative control. Tissue sections of liver and lung were prepared for H&E staining. Scale bar, 100 μm.

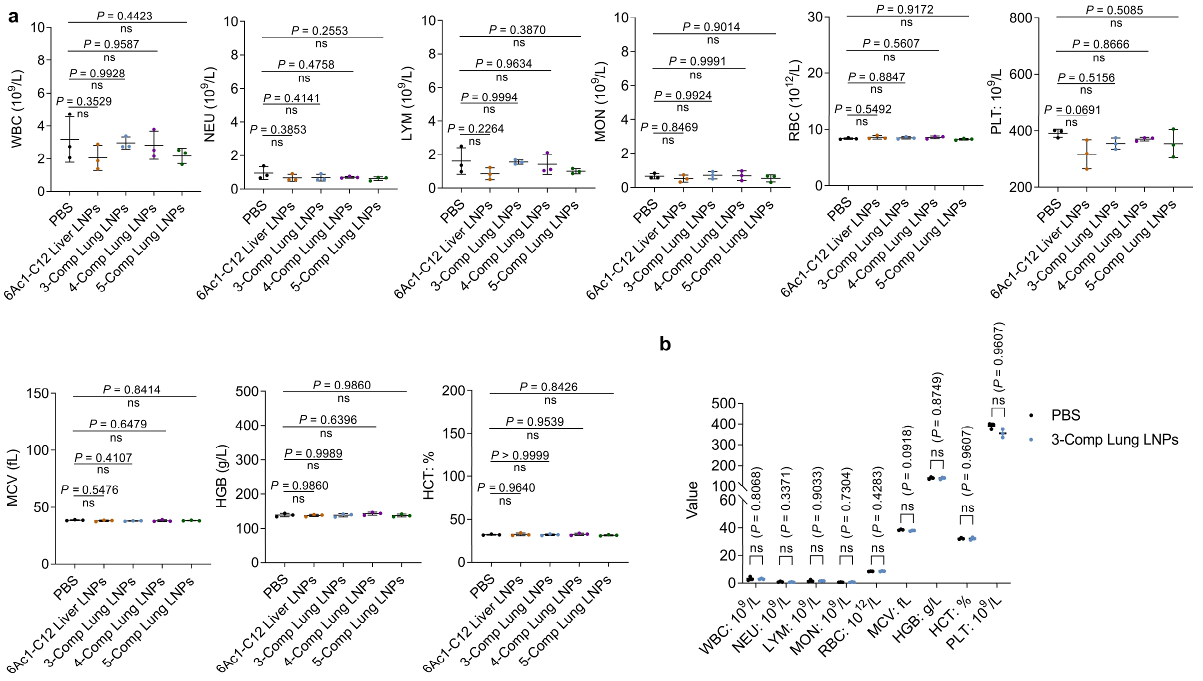

Supplementary Fig. 34 | nAcx-Cm Liver and Lung LNPs were well tolerated in vivo. nAcx-Cm Liver and Lung LNPs were i.v. administrated to C57BL/6 mice. PBS (i.v.) was set as the negative control. **a**, Major hematological analysis was performed 24 h post administration. The main hematological analysis included assessments for white blood cell (WBC), neutrophil (NEU), lymphocyte (LYM), monocyte (MON), red blood cell (RBC), platelet (PLT), mean corpuscular volume (MCV), hemoglobin (HGB), and hematocrit (HCT). There was no significant difference between the nAcx-Cm LNPs and PBS groups. **b**, The major hematological analysis of 3-Comp Lung LNPs showed no significant differences compared to the negative control. All data are presented as mean ± s.d. (n = 3 biologically independent animals). Statistical significances in **a** were calculated using one-way ANOVA with Dunnett's multiple comparisons test, and those in **b** were calculated with a two-tailed unpaired t-test: $^{****}P < 0.0001$; $^{***}P < 0.001$; $^{**}P < 0.01$; $^{*}P < 0.05$; ns, no significant difference.

Supplementary References

1. Liu, S. *et al.* Membrane-destabilizing ionizable phospholipids for organ-selective mRNA delivery and CRISPR–Cas gene editing. *Nat. Mater.* **20**, 701–710 (2021).
2. Zhou, K. *et al.* Modular degradable dendrimers enable small RNAs to extend survival in an aggressive liver cancer model. *Proc. Natl Acad. Sci. USA* **113**, 520–525 (2016).
3. Bourgognon, M., Klippstein, R. & Al-Jamal, K. T. Kupffer cell isolation for nanoparticle toxicity testing. *J. Vis. Exp.* 52989 (2015).
