# Supplementary material for: Reformulating lipid nanoparticles for organ-targeted mRNA accumulation and translation
Source: Nat Commun. 2024 Jul 5;15:5659. doi: 10.1038/s41467-024-50093-7 (PMC11226454; doi:10.1038/s41467-024-50093-7)
Supplement: Supplementary file 3 — Reporting Summary [file 41467_2024_50093_MOESM3_ESM.pdf]

nature portfolio

Corresponding author(s): Shuai Liu, Zhen Gu, Yuan Ping

Last updated by author(s): Jun 24, 2024

Reporting Summary

Nature Portfolio wishes to improve the reproducibility of the work that we publish. This form provides structure for consistency and transparency in reporting. For further information on Nature Portfolio policies, see our Editorial Policies and the Editorial Policy Checklist.

Statistics

For all statistical analyses, confirm that the following items are present in the figure legend, table legend, main text, or Methods section.

n/a	Confirmed	
[ ]	[x]	The exact sample size (*n*) for each experimental group/condition, given as a discrete number and unit of measurement
[ ]	[x]	A statement on whether measurements were taken from distinct samples or whether the same sample was measured repeatedly
[ ]	[x]	The statistical test(s) used AND whether they are one- or two-sided<br>*Only common tests should be described solely by name; describe more complex techniques in the Methods section.*
[x]	[ ]	A description of all covariates tested
[x]	[ ]	A description of any assumptions or corrections, such as tests of normality and adjustment for multiple comparisons
[ ]	[x]	A full description of the statistical parameters including central tendency (e.g. means) or other basic estimates (e.g. regression coefficient) AND variation (e.g. standard deviation) or associated estimates of uncertainty (e.g. confidence intervals)
[ ]	[x]	For null hypothesis testing, the test statistic (e.g. *F*, *t*, *r*) with confidence intervals, effect sizes, degrees of freedom and *P* value noted<br>*Give P values as exact values whenever suitable.*
[x]	[ ]	For Bayesian analysis, information on the choice of priors and Markov chain Monte Carlo settings
[x]	[ ]	For hierarchical and complex designs, identification of the appropriate level for tests and full reporting of outcomes
[x]	[ ]	Estimates of effect sizes (e.g. Cohen's *d*, Pearson's *r*), indicating how they were calculated

Our web collection on statistics for biologists contains articles on many of the points above.

Software and code

Policy information about availability of computer code

Data collection	ZS Xplorer software version 3.3.0.42 (Malvern Panalytical)<br>ZEN x64 software version 1.1.0 (Carl Zeiss Microscopy GmbH)<br>FV31S-SW software version 2.3.2.169 (Olympus Corporation)<br>Living Image software version 4.7.4 (64-bit, Caliper Life Sciences)<br>BD FACSDiva software version 8.0.3 (BD LSRFortessa)<br>Pannoramic scanner (3DHISTECH)<br>Automatic Blood Analyzer (TECOM)
Data analysis	ZEN 2010 software version 6.0.62 (Carl Zeiss Microscopy GmbH)<br>GraphPad Prism 9 software version 9.0.0 (GraphPad Software)<br>Living Image software version 4.7.4 (64-bit, Caliper Life Sciences)<br>FLOWJO software version 10.8.1 (FLOWJO)<br>CaseViewer 2.4 (3DHISTECH)

For manuscripts utilizing custom algorithms or software that are central to the research but not yet described in published literature, software must be made available to editors and reviewers. We strongly encourage code deposition in a community repository (e.g. GitHub). See the Nature Portfolio guidelines for submitting code & software for further information.

Data

Policy information about availability of data

All manuscripts must include a data availability statement. This statement should provide the following information, where applicable:

- Accession codes, unique identifiers, or web links for publicly available datasets
- A description of any restrictions on data availability
- For clinical datasets or third party data, please ensure that the statement adheres to our policy

All data supporting the findings of this study are presented in the Article, Supplementary Information, and Source Data file. The data that support the findings of this study are available from the corresponding author upon reasonable request. Source data are provided with this paper. Source data is available for Figures 2-5 and Supplementary Figures 3-7, 9, 17, 19, 22, 25, 26, 31, 32 and 34 in the associated source data file.

Research involving human participants, their data, or biological material

Policy information about studies with human participants or human data. See also policy information about sex, gender (identity/presentation), and sexual orientation and race, ethnicity and racism.

Reporting on sex and gender	Not applicable.
Reporting on race, ethnicity, or other socially relevant groupings	Not applicable.
Population characteristics	Not applicable.
Recruitment	Not applicable.
Ethics oversight	Not applicable.

Note that full information on the approval of the study protocol must also be provided in the manuscript.

Field-specific reporting

Please select the one below that is the best fit for your research. If you are not sure, read the appropriate sections before making your selection.

☒ Life sciences ☐ Behavioural & social sciences ☐ Ecological, evolutionary & environmental sciences

For a reference copy of the document with all sections, see nature.com/documents/nr-reporting-summary-flat.pdf

Life sciences study design

All studies must disclose on these points even when the disclosure is negative.

Sample size	Sample sizes were determined using statistical power calculations. For various assays, we performed at least three replicates to yield statistically significant differences.
Data exclusions	No data were excluded from the analysis.
Replication	We confirm that all results reported in the paper were replicated successfullly across multiple experiments. We have used C57BL/6 mice and tdTomato reporter mice (Ai9 mice) to confirm tissue specific mRNA delivery. The narrow variations also confirmed that our experimental findings are reproducible. Key data generated by one co-author were repeated by other co-authors.
Randomization	For animal experiments, mice with ages of 6-8 weeks were randomly allocated into each treatment group.
Blinding	Due to the proof-of-concept developmental nature of this study, true blinding of experiments was not performed. However, data collection and analysis for some experiments were performed by separate individuals. In some cases, these collectors/analyzers were not aware which samples corresponded to which experimental groups at the time of data collection and analysis.

Reporting for specific materials, systems and methods

We require information from authors about some types of materials, experimental systems and methods used in many studies. Here, indicate whether each material, system or method listed is relevant to your study. If you are not sure if a list item applies to your research, read the appropriate section before selecting a response.

Materials & experimental systems

n/a	Involved in the study	
☐	☒	Antibodies
☐	☒	Eukaryotic cell lines
☒	☐	Palaeontology and archaeology
☐	☒	Animals and other organisms
☒	☐	Clinical data
☒	☐	Dual use research of concern
☒	☐	Plants

Methods

n/a	Involved in the study	
☒	☐	ChIP-seq
☐	☒	Flow cytometry
☒	☐	MRI-based neuroimaging

Antibodies

Antibodies used

We used multiple monoclonal antibodies in the flow cytometry to determine tissue specific accumulation in C57BL/6 mice and tissue specific editing by Cre mRNA in tdTomato mice in Fig.5 and SI figures. These antibodies are: PerCP/Cyanine5.5 anti-mouse CD45 (Biolegend, 157208), PE/Cyanine7 anti-mouse CD31 (Biolegend, 102524), FITC anti-mouse/human CD11b (Biolegend, 101205), Brilliant Violet 785 anti-mouse F4/80 (Biolegend, 123141), FITC anti-mouse CD326 (Ep-CAM) (Biolegend, 118207), APC anti-mouse F4/80 (Biolegend, 101205), APC anti-mouse CD45 (Biolegend, 103112).

Validation

1. PerCP/Cyanine5.5 anti-mouse CD45 has been validated to be used for immunofluorescent staining with flow cytometric analysis. The suggested use of this reagent is ≤ 0.25 µg per million cells in 100 µL volume from the manufacturer's website and it is also mentioned species reactivity with mouse. (https://www.biolegend.com/en-gb/products/percpcyanine55-anti-mouse-cd45-antibody-19248). After titration, we finally used 1/200 dilution for liver and lung cell types.
2. PE/Cyanine7 anti-mouse CD31 has been validated to be used for immunofluorescent staining with flow cytometric analysis. The suggested use of this reagent is ≤0.125 µg per million cells in 100 µL volume from the manufacturer's website and it is also mentioned species reactivity with mouse. (https://www.biolegend.com/en-gb/products/pe-cyanine7-anti-mouse-cd31-antibody-12996). After titration, we finally used 1/200 dilution for liver and lung cell types.
3. FITC anti-mouse/human CD11b has been validated to be used for immunofluorescent staining with flow cytometric analysis. The suggested use of this reagent is ≤ 0.25 µg per million cells in 100 µL volume from the manufacturer's website and it is also mentioned species reactivity with mouse. (https://www.biolegend.com/en-gb/products/fitc-anti-mouse-human-cd11b-antibody-347). After titration, we finally used 1/100 dilution for liver cell types.
4. Brilliant Violet 785 anti-mouse F4/80 has been validated to be used for immunofluorescent staining with flow cytometric analysis. The suggested use of this reagent is ≤0.5 µg per million cells in 100 µL volume from the manufacturer's website and it is also mentioned species reactivity with mouse. (https://www.biolegend.com/en-gb/products/brilliant-violet-785-anti-mouse-f4-80-antibody-9919). After titration, we finally used 1/100 dilution for liver cell types.
5. FITC anti-mouse CD326 (Ep-CAM) has been validated to be used for immunofluorescent staining with flow cytometric analysis. The suggested use of this reagent is ≤ 0.25 µg per million cells in 100 µL volume from the manufacturer's website and it is also mentioned species reactivity with mouse. (https://www.biolegend.com/en-gb/products/fitc-anti-mouse-cd326-ep-cam-antibody-4971). After titration, we finally used 1/100 dilution for lung cell types.
6. APC anti-mouse F4/80 has been validated to be used for immunofluorescent staining with flow cytometric analysis. The suggested use of this reagent is ≤ 0.25 µg per million cells in 100 µL volume from the manufacturer's website and it is also mentioned species reactivity with mouse.(https://www.biolegend.com/en-gb/products/apc-anti-mouse-f4-80-antibody-4071). After titration, we finally used 1/100 dilution for liver cell types.
7. APC anti-mouse CD45 has been validated to be used for immunofluorescent staining with flow cytometric analysis. The suggested use of this reagent is ≤ 0.25 µg per million cells in 100 µL volume from the manufacturer's website and it is also mentioned species reactivity with mouse.(https://www.biolegend.com/en-gb/products/apc-anti-mouse-cd45-antibody-97). After titration, we finally used 1/200 dilution for lung cell types.

Eukaryotic cell lines

Policy information about cell lines and Sex and Gender in Research

Cell line source(s)

IGROV1 cells were originally obtained from ATCC.

Authentication

The cell lines were not further authenticated after receiving.

Mycoplasma contamination

The cell lines were not tested for mycoplasma contamination.

Commonly misidentified lines
(See ICLAC register)

No commonly misdentified cell lines were used.

Animals and other research organisms

Policy information about studies involving animals; ARRIVE guidelines recommended for reporting animal research, and Sex and Gender in Research

Laboratory animals

Mice were maintained in a barrier facility with a 12-h light/12-h dark cycle, at around 20 °C and 40% humidity. C57BL/6 mice were obtained from GemPharmatech Co., Ltd. B6.Cg-Gt(ROSA)26Sortm9(CAGtdTomato)Hze/J mice (also known as Ai9 or Ai9(RCL-tdT)

	mice) were obtained from The Jackson Laboratary (007909) and bred to maintain homozygous expression of the Cre reporter allele that has a loxP-flanked STOP cassette preventing transcription of a CAG promoter-driven red fluorescent tdTomato protein. Following Cre-mediated recombination, Ai9 mice will express tdTomato fluorescence. Ai9 mice are congenic on the C57BL/6J genetic background. Ai9 male or female mice with ages of 6-8 weeks were used.
Wild animals	The study did not involve wild animals.
Reporting on sex	Both male and female mice were used. The sex of the animals was not further considered because LNP-mediated nucleic acid delivery data did not show bias toward one gender in experiments.
Field-collected samples	The study did not involve samples collected from the field.
Ethics oversight	All animal handing procedure were approve by Laboratory Animal Welfare and Ethics Committee of Zhejiang University.

Note that full information on the approval of the study protocol must also be provided in the manuscript.

Plants

Seed stocks	*Report on the source of all seed stocks or other plant material used. If applicable, state the seed stock centre and catalogue number. If plant specimens were collected from the field, describe the collection location, date and sampling procedures.*
Novel plant genotypes	*Describe the methods by which all novel plant genotypes were produced. This includes those generated by transgenic approaches, gene editing, chemical/radiation-based mutagenesis and hybridization. For transgenic lines, describe the transformation method, the number of independent lines analyzed and the generation upon which experiments were performed. For gene-edited lines, describe the editor used, the endogenous sequence targeted for editing, the targeting guide RNA sequence (if applicable) and how the editor was applied.*
Authentication	*Describe any authentication procedures for each seed stock used or novel genotype generated. Describe any experiments used to assess the effect of a mutation and, where applicable, how potential secondary effects (e.g. second site T-DNA insertions, mosiacism, off-target gene editing) were examined.*

Flow Cytometry

Plots

Confirm that:

- [x] The axis labels state the marker and fluorochrome used (e.g. CD4-FITC).
- [x] The axis scales are clearly visible. Include numbers along axes only for bottom left plot of group (a 'group' is an analysis of identical markers).
- [x] All plots are contour plots with outliers or pseudocolor plots.
- [x] A numerical value for number of cells or percentage (with statistics) is provided.

Methodology

Sample preparation	To isolate mouse liver cells, separation was conducted by differential centrifugation. Mice were anesthetized with isoflurane and fixed. The mouse liver was perfused, and then cut and digested with collagenase IV (5 mL) at 37 °C for 30 min. After terminating the digestion, the liver cells were passed through a 70 µm cell filter and washed with 1× PBS. Liver parenchyma cells were collected by centrifugation (50 g, 5 min), and the cell precipitate was resuspended in the washing medium and washed with 1× PBS. The supernatant was collected by centrifugation (450 g, 5 min) to give liver non-parenchymal cells. These cells were resuspended in the red blood lysis buffer and incubated for 5 min, then 1× PBS was added to terminate the lysis. The mixture was centrifuged, counted, and resuspended in the cell staining buffer. Following this, staining antibodies were added, and the cells were incubated on ice for 30 min in the dark. The cells were then washed twice with 1× PBS and finally resuspended in 500 µL of 1× PBS. The cell suspension was transferred to the flow tubes and analyzed using a multicolor analytical flow analyzer (LSR Fortessa, BD Biosciences).<br>To isolate and stain lung cells, the lung was minced and added to a 15 mL tube containing Collagenase I and DNase I. The mixture was incubated at 37 °C for 1 h. Following termination of digestion, the mixture was filtered through a 70 µm cell filter and washed with 1× PBS. The remaining steps were consistent with the protocol of liver cell collection mentioned above. Ultimately, lung cells were analyzed using the LSRForessa machine described above. Data were analyzed using FLOWJO software version 10.8 (FLOWJO).
Instrument	LSRFortessa (BD Biosciences)
Software	Data collection: BD FACSDiva software version 8.0.3 (BD LSRFortessa);<br>Data analysis: FLOWJO software version 10.8.1 (FLOWJO)
Cell population abundance	We used C57BL/6 mice to evaluate tissue biodistribution via detecting the Cy5 mean fluorescence intensity. We used tdTomato (Ai9) mice to evaluate tissue specific gene editing efficiency (Cre mediated) via detecting the tdTomato mean fluorescence intensity.

Gating strategy

Gates for Cy5+ and td-Tomato+ in cell types were drawn based on control mice. Gating strategy were provided in SI Figures.

☒ Tick this box to confirm that a figure exemplifying the gating strategy is provided in the Supplementary Information.
